# The Panda-Derived *Lactobacillus plantarum* G201683 Alleviates the Inflammatory Response in DSS-Induced Panda Microbiota-Associated Mice

**DOI:** 10.3389/fimmu.2021.747045

**Published:** 2021-12-08

**Authors:** Yi Zhou, Ling Duan, Yan Zeng, Lili Niu, Yang Pu, Jonathan P. Jacobs, Candace Chang, Jie Wang, Abdul Khalique, Kangcheng Pan, Jing Fang, Bo Jing, Dong Zeng, Xueqin Ni

**Affiliations:** ^1^ Animal Microecology Research Center, College of Veterinary Medicine, Sichuan Agricultural University, Chengdu, China; ^2^ Central Station of Animal Feed Affairs of Sichuan Province, Sichuan Provincial Department of Agriculture and Rural Affairs, Chengdu, China; ^3^ Chengdu Wildlife Institute, Chengdu Zoo, Chengdu, China; ^4^ The Vatche and Tamar Manoukian Division of Digestive Diseases, Department of Medicine, David Geffen School of Medicine at University of California, Los Angeles (UCLA), Los Angeles, CA, United States; ^5^ Division of Cardiology, Department of Medicine, David Geffen School of Medicine at University of California, Los Angeles (UCLA), Los Angeles, CA, United States

**Keywords:** giant panda, inflammation, *Lactobacillus*, microbiota, intestinal barrier

## Abstract

Intestinal diseases are one of the main causes of captive giant panda death. Their special dietary habits and gastrointestinal tract structure often lead to intestinal epithelium damage and secondary intestinal infection. The captive giant panda is predisposed to suffer from microbiota dysbiosis due to long-term artificial feeding and antibiotic misuse. However, there are few reported probiotics to treat giant panda enteritis and the associated dysbiosis. This study aims to elucidate the mechanism by which *Lactobacillus plantarum* G201683 (*L. plantarum* G83), a promising panda-derived probiotic, exerts a protective effect on intestinal inflammation in the dextran sulfate sodium- (DSS) induced panda microbiota-associated (DPMA) mouse model. The DPMA mouse was generated by antibiotic treatment and 5% DSS drinking water administration to assess the effect of *L. plantarum* G83 on intestinal inflammation and microbiota *in vivo*. Our results demonstrated the successful generation of a DPMA mouse model with *Enterobacteriaceae* enrichment, consistent with the giant panda intestinal microbiota. *L. plantarum* G83 decreased clinical and histological severity of intestinal inflammation, enhanced intestinal tight junction protein expression (ZO-1, Occludin) and alleviated inflammatory cytokine production (TNF-) in the colon of DPMA mice. The administration of *L. plantarum* G83 altered the microbiota composition by decreasing pathogen associated taxa such as *E. coli* and increasing abundance of beneficial bacteria including *Bifidobacterium* spp. These changes in microbiota composition were associated with an increased concentration of short chain fatty acids (SCFA), reduced NF-κB signaling, and an altered balance of T helper cell subsets. Our findings support *L. plantarum* G83 as a promising probiotic to treat intestinal inflammation in the giant panda.

**Graphical Abstract:**
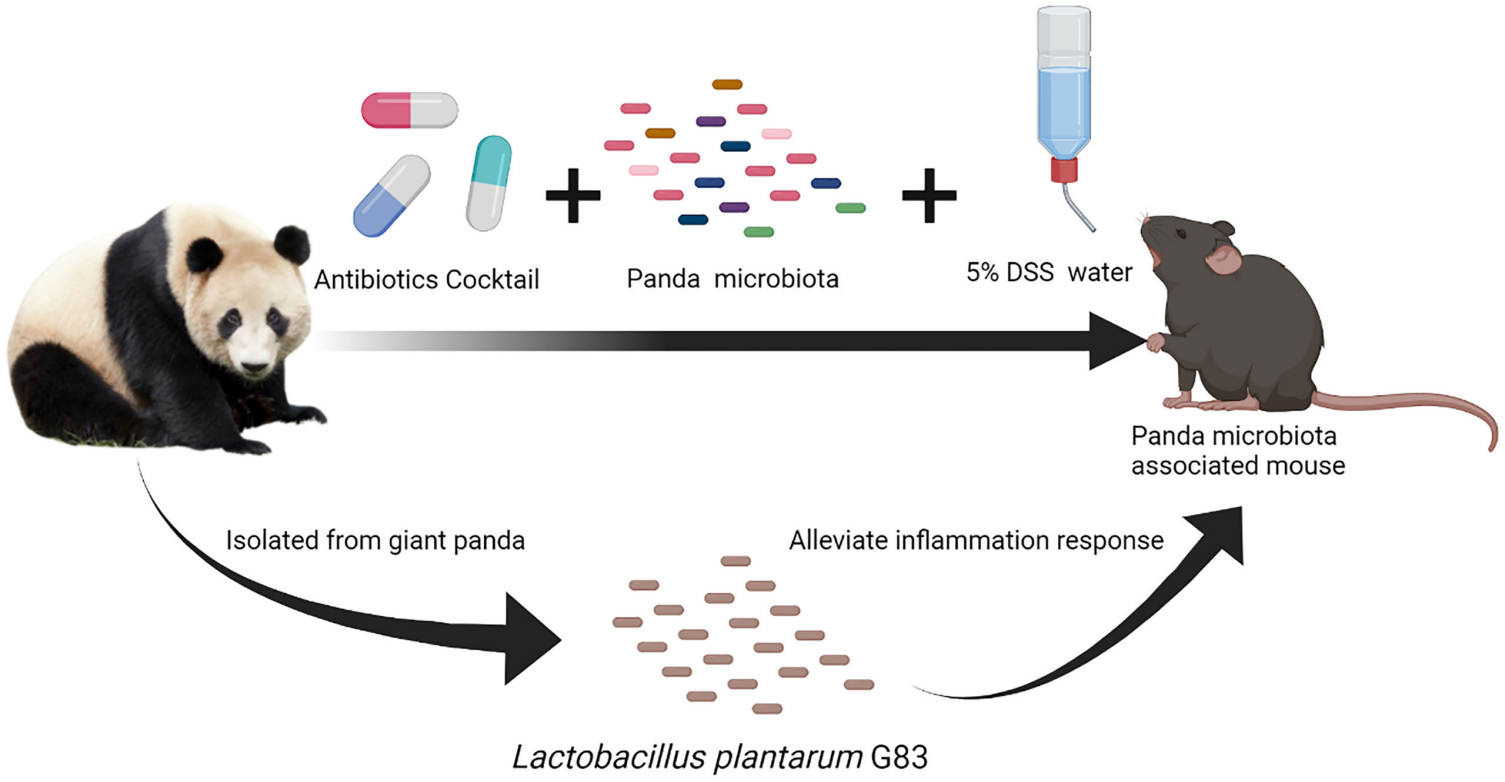


## Introduction

The giant panda (*Ailuropoda melanoleuca*), belonging to the carnivorous family Ursidae, is the “flagship” animal of the World Wildlife Fund. Although giant pandas have a carnivore-like gut microbiota, they are actually “vegetarians” and rely on bamboo as their main diet (1). They also maintain a distinctive intestinal microbiota dominated by *Streptococcus*, *Clostridium*, and *Enterobacteriaceae* (2). However, these predominant species are commonly observed in intestinal diseases; in particular, many reported intestinal pathogens belong to *Enterobacteriaceae*. The intestinal tract of giant pandas is readily damaged by undigested bamboo fibers and parasitic infection. Even more regrettably, a large number of opportunistic pathogens from *Enterobacteriaceae* often cause secondary infections and lead to intestinal inflammation (3, 4). While antibiotics are widely used to treat intestinal infections in the giant panda, recent studies have demonstrated widespread antibiotic resistance among fecal isolates of *Escherichia coli* (*E. coli*), a prominent member of the Enterobacteriacae family. In one study, 89 *E. coli* isolates from panda feces (5) showed drug resistance with a frequency of 54 to 93% resistance to individual antibiotics. In a second study, 31 tetracycline-resistant and 11 amoxicillin-resistant strains were found in 88 isolated *E. coli* from 61 different feces samples (6). Widespread drug-resistance is a plausible threat to pandas as well as other captive animals.

There is considerable evidence that the consumption of probiotics can benefit mammalian health. Probiotics act on host health through multiple possible ways, namely, intestinal epithelial barrier maintenance and immune modulation (7). Intestinal epithelial tight junction (TJ) proteins act as a functional and structural barrier against the paracellular permeability of luminal antigens (8). The *Lactobacillus* spp. are suggested to prevent the development of intestinal inflammation by enhancing TJ expression and adjusting the distribution of TJ proteins, strengthening the intestinal barrier. Several studies indicate that *L.* casei (9), *L. plantarum* (10), and *L. rhamnosus* (11) cause modest enhancements in intestinal epithelial TJ proteins. There are many inflammatory pathways involved in bacterial infection that may be targeted by *Lactobacillus* spp. to regulate intestinal tight junction proteins, helping TJ assembly and redistribution on the cell surface (12). In addition, the *Lactobacillus* genus is known to act as an active immune mediator by interacting with many antigen-presenting cells (APCs) in the intestine (13). Intestinal immune cells can regulate immune response induced by microbe-associated molecular patterns (MAMPs) through pattern recognition receptors (PRRs) such as Toll-like receptors (TLRs) (14, 15). Many studies demonstrated that *Lactobacillus* regulates immune processes by inducing TLR2 and TLR4 dependent pathways to boost or inhibit pro-inflammatory cytokine release (15–17). Furthermore, *Lactobacillus* has been shown to modulate maturation of dendritic cells (DCs), thereby promoting T helper (Th) cell differentiation (18, 19). *Lactobacillus* has been reported to ameliorate DSS and TNBS colitis by enhancing CD4^+^FoxP3^+^ regulatory T cells in mesenteric lymph nodes and decreasing pro-inflammatory cytokines in Peyer’s patches by regulating Th1-Th2-Th17 cell balance (20–23).

It has been demonstrated that *Lactobacillus* strains interact with other gut microbes to support microbiota stability, making it a good candidate to mitigate gut microbiota dysbiosis (24). *Lactobacillus* can also effectively compete with pathogens for ecological niches within the gut. For example, *L. plantarum* 0612 has been reported to outcompete *E. coli* ATCC 11775 and *Listeria monocytogenes* ATCC 13932 for cell surface adhesion *in vitro* and *in vivo*, and the abundance of enteropathogenic bacteria was significantly decreased after *L. plantarum* 0612 supplementation (25). Moreover, organic acids, the primary end products of *Lactobacillus* metabolism, not only contribute to lower luminal pH and inhibit the growth of pathogens but also act as an intermediate to cross-feed commensal bacteria (26). For instance, *Lactobacillus* do not directly produce butyrate but cross-feed butyrate-producing commensal microbiota by providing acetate (27–29).

There are few reports, however, about the protective effects of probiotics on intestinal diseases in giant panda, and most studies focus on cellulose/hemicellulose metabolism. In our previous studies, the *L. plantarum* G201683 (*L. plantarum* G83) strain isolated from giant panda feces showed a strong antagonistic action against pathogenic bacteria *in vitro* and an ability to alleviate inflammation caused by enterotoxigenic *E. coli* infection *in vivo* (30, 31). Therefore, we hypothesize that *L. plantarum* G201683 ameliorates intestinal inflammatory diseases in giant pandas. To address this hypothesis, we generated a DSS-induced giant panda microbiota-associated (DPMA) mouse model by fecal microbiota transplantation and dextran sulfate sodium (DSS) oral administration. This murine model allowed us to assess the effect of *L. plantarum* G201683 on the inflammatory response and panda-associated microbiota.

## Material and Method

### *L. plantarum* G83 Strain

The *L. plantarum* G83 (CCTCC M2016245) was isolated from giant panda feces. It is available at the China Center for Type Culture Collection (CCTCC, Wuhan, CHN).

### The Giant Panda Feces Microbiota Inoculum

The feces samples were collected from three healthy sub-adult pandas (Chengdu Research Base of Giant Panda Breeding, CHN) and mixed. The fecal inoculum was prepared as described by Zeng (32). In brief, 140 ml pre-reduced PBS was added into 60 g mixed fresh fecal sample. The inoculum was homogenized by vortex mixer. Thereafter, the inoculum was sequentially passed through 2.0-, 1.0- and 0.5-mm diameter stainless steel strainers to remove larger particles and to concentrate filtrate to 10 ml. The giant panda microbiota inoculum (PFM) was prepared before gavage daily.

### The Giant Panda Microbiota-Associated (PMA) Mouse and DSS-Induced Panda Microbiota-Associated Mouse (DPMA Mouse)

The experiment was completed under the supervision of the Institutional Animal Care and Use Committee of the Sichuan Agricultural University. Seven-week-old female SPF-C57BL/6J mice, purchased from the Beijing Vital River Laboratory Animal Technology Co. Ltd, were separated into five groups equally. Three mice were housed per cage and provided with free access to sterile distilled water and food. One group of mice was set up as a control group (Blank). The remaining mice were provided sterile water with ampicillin 600 mg/L, vancomycin 600 mg/L, and neomycin 600 mg/L from days 1 to 7. After the antibiotics cocktail treatment, two different methods (A and B) were used to generate the giant panda microbiota-associated (PMA) mouse (**Supplementary Figures 1A, B**). In method A, the mice were gavaged with 0.2 ml/day panda feces microbiota inoculum three times (on days 8, 9, and 10) after sterile water was provided (PMA mouse, BlankT group). We continued monitoring the body weight from days 0 to 13. Fecal pellets were collected on days 0, 7, 10 and 13. In method B, the mice were gavaged with the microbiota inoculum as mentioned in method A, followed by the administration of 5.0% (w/v) DSS (molecular mass 3.6–5.0 kDa; MP Biomedical, Irvine, CA, USA) in drinking water for 3 days from days 11 to 13 (DPMA mouse, BlankD group). We monitored the body weight of the subjects from days 0 to 16. Fecal pellets were collected on days 1, 7, 13, and 16.

### Animal Trials

DPMA mice were prepared following the method-B mentioned above, then the mice were separated into five different groups (**Supplementary Figure 1C**): 1) the control group (no treatment received, BlankD), 2) mesalazine treatment group (94 mg/d, switched into the equivalent dose to the mouse, TreatMz group), and 3) three 0.2 ml/d *L. plantarum* G83 treatment groups [namely, 1.0 × 10^6^ CFU/ml (TreatLG group), 1.0 × 10^7^ CFU/ml (TreatMG group), 1.0 × 10^8^ CFU/ml (TreatHG group)] from days 14 to 16. On day 16, the mice were euthanized and the blood, colon, spleen, payer’s patches, mesenteric lymph nodes, and colon content were collected for further analysis. Fecal pellets were collected on days 1, 7, 13, and 16. We monitored the body weight and the disease activity score from days 1 to 16.

### Viable Bacterial Count

The fecal pellets were serially diluted from 0 to 10^−6^ in 10-fold and the diluted samples were plated on BHI (for total bacteria), TPY (for *Bifidobacteria*), EC (for *Enterococcus*), EMB (for *Escherichia coli*), and MRS (for *Lactobacillus*) agar. EC and EMB agar plates were maintained in the aerobic incubator, while the TPY agar was incubated in an anaerobic chamber for anaerobic microorganisms. One BHI plate was maintained aerobically and the another was maintained anaerobically. Following incubation, colony-forming units (CFU) counts were performed the next day.

### Western Blot

The protein lysate was obtained from tissue by culture cell total protein extraction reagent (BOSTER, Wuhan, CHN) and mammalian tissue protein extraction reagent (BOSTER, Wuhan, CHN), with protease inhibitor and phosphatase inhibitor cocktail, respectively. Protein concentration was determined by a BCA protein analysis kit (BIOMED, Beijing, CHN). The primary antibodies included the following: rabbit anti-β-actin (#20536-1-AP; Proteintech, Wuhan, CHN), rabbit anti-ZO-1 (#21773-1-AP; Proteintech, Wuhan, CHN), rabbit anti-occludin (#27260-1-AP; Proteintech, Wuhan, CHN), rabbit anti-Claudin 1 (#bs-1428R; Bioss, Beijing, CHN), rabbit anti-NF-κB P65 (#bs-0465R; Bioss, Beijing, CHN), rabbit anti-phospho-NF-κB p65 (#3033T; CST, Danvers, MA USA), rabbit anti-p38 (#ab27986; Abcam, Cambridge, MA, USA), and rabbit anti-phospho-p38(#ab47363; Abcam, Cambridge, MA, USA). The secondary antibody used was goat anti-rabbit (#bs-0296G; Bioss, Beijing, CHN).

### Histology

The colon tissue samples were fixed in 4% paraformaldehyde fix solution and embedded in paraffin. Sections (5-μm thick) were stained with hematoxylin and eosin (HE). Colonic epithelial slices were scored following Kyoko’s method (33). The score included epithelial damage and inflammatory cell infiltration, and the total score ranged from 0 to 12. For immuno-histochemical staining, the tissues were processed following the conventional method. Subsequently, the primary antibody was added to the slide and incubated overnight at 4°C in a histochemistry staining tray. The HRP-conjugated secondary antibody was then added for 50 min, and the DAB was added for 3 min. Finally, the slides were counterstained with hematoxylin, dehydrated, and sealed for visualization. The images were captured using an optical microscope (Olympus, Tokyo, Japan), and the positively stained cells were scored by ImageJ (NIH, Stapleton, NY, USA).

### Flow Cytometry

Flow cytometry was performed to measure the following: CD3^+^ T cells (PerCP/Cy5.5 anti-mouse CD3, clone 17A2, Biolegend, San Diego, CA, USA), CD4^+^ T cells (CD3^+^ CD4^+^, FITC anti-mouse CD4, clone RM4-5, Biolegend, San Diego, CA, USA), CD8^+^ T cells (CD3^+^CD8^+^, PE anti-mouse CD8a, clone 53-6.7, Biolegend, San Diego, CA, USA). Th1 (PerCP-Cy5.5 anti-mouse CD4, clone RM4-5, FITC anti-mouse IFN-GMA, clone XMG1.2, BD Biosciences, San Jose, CA, USA), Th2 (APC anti-mouse IL-4, clone 11B11, BD Biosciences, San Jose, CA, USA), and Th17 (PE anti-mouse IL-17A, clone TC11-18H10.1, BD Biosciences, San Jose, CA, USA) phenotypes were also characterized. Lymphocytes were collected from the peripheral blood (PB), Peyer’s patches (PPs), mesenteric lymph nodes (MLNs), and spleen on day 16. In the surface phenotype assay, 1.0 × 10^6^ cells were blocked with 10 μl rat serum for 30 min at 4°C and stained with the indicated antibody for 30 min at 4°C in the dark. Cells for the intracellular cytokine assay were stimulated with phorbol 12-myristate 13-acetate (MilliporeSigma, Burlington, MA, USA), monensin (MilliporeSigma, Burlington, MA, USA), and ionomycin (MilliporeSigma, Burlington, MA, USA) for 4 h. The cells were labeled with surface markers, before being fixed, permeabilized, and labeled with the indicated intracellular antibody for 30 min at 4°C in the dark. Cells were acquired on the BD FACS-Verse™ flow cytometer (BD Biosciences, San Jose, CA, USA) and the data was analyzed using the FlowJo 10.0.7 software (BD Biosciences, San Jose, CA, USA).

### Short-Chain Fatty Acids (SCFAs)

About 0.7 g of the colonic content (mass per sample recorded) was added in a 2 ml centrifuge tube with 1.5 ml ultrapure water. It was thoroughly mixed, set aside for 30 min, and centrifuged at 4°C, 12,000×*g*, for 15 min. Approximately 1 ml supernatant was mixed with 0.2 ml metaphosphoric acid solution [25% (w/v)] and 23.3 μl crotonic acid solution (210 mmol/L) before being centrifuged at 4°C, 12,000×*g* for 10 min after incubation at 4°C for 30 min. Following centrifugation, 0.3 ml of supernatant was added to 0.9 ml of chromatographic methanol (1:3 dilution) and centrifuged at 4°C, 12,000×*g*, for 5 min. The supernatant was filtered through 0.22 μm syringe filter. Shimadzu 2010 Plus AF gas chromatograph was used to determine SCFAs.

### RNA Extraction and Quantitative Real-Time PCR (q-PCR)

The total RNA was isolated from colon tissue using TRIzol Reagent (Invitrogen, Carlsbad, CA, USA). The first-strand cDNA was synthesized from the total RNA using RevertAid First Strand cDNA Synthesis Kit (ThermoFisher, Waltham, MA, USA). The q-PCR was performed using iTaq™ Universal SYBR Green on the CFX Connect™ Real-Time PCR Detection System (Bio-Rad, Hercules, CA, USA). Primers and annealing temperature are shown in **Supplementary Table 1**. The result of relative gene expression was normalized to that of β-actin and evaluated through the 2^−△△Ct^ method.

### Microbiota DNA Extraction and Sequencing

The total microbial DNA was obtained from the colon content or fecal pellets by E.Z.N.A.^®^ Stool DNA Kit (OMEGA, Norcross, GA, USA) and quantified by NanoDrop™ 2000 Spectrophotometer (ThermoFisher, Waltham, MA, USA). PCR amplification of V3–V4 regions of bacterial 16S rRNA genes was performed using universal primer sequences 341F (5’-barcode-CCTAYGGGRBGCASCAG) and 806R (5’-barcode-GGACTACNNGGGTATCTAAT). The PCR products were purified by QIAquick Gel Extraction Kit (Qiagen, Germantown, MD, USA). Thereafter, the amplicon libraries were prepared using Ion Plus Fragment Library Kit (ThermoFisher, Waltham, MA, USA) and the sequencing was performed on Ion S5™ XL system (ThermoFisher, Waltham, MA, USA).

### 16S rRNA Sequence Analysis

Data were analyzed using Novogene cloud analysis platform (https://magic.novogene.com). Raw FASTQ files were edited to exclude adaptor contamination (Cutadapt, V1.9.1, http://cutadapt.readthedocs.io/en/stable/). Chimeras were detected using UCHIME Algorithm (http://www.drive5.com/usearch/manual/uchime_algo.html). The clean reads were clustered into operational taxonomic units (OTUs) by Uparse software (v7.0.1001, http://www.drive5.com/uparse/) at 97% identity. Alpha diversity and beta diversity were evaluated using QIIME (Version 1.9.1). Differentially abundant taxa were identified using linear discriminant analysis (LDA) effect size (LEfSe) (https://huttenhower.sph.harvard.edu/lefse/). Functional annotation of prokaryotic taxa (FAPROTAX) was performed to identify ecologically relevant functions based on the current literature on cultured strains (https://pages.uoregon.edu/slouca/LoucaLab/archive/FAPROTAX/lib/php/index.php). DESeq2 was used to detect differentially abundant microbes at the genus level (https://bioconductor.org/packages/release/bioc/html/DESeq2.html).

## Result

### Microbial Composition and Diversity of Panda-Microbiota Associated Mice

**Figure 1:**
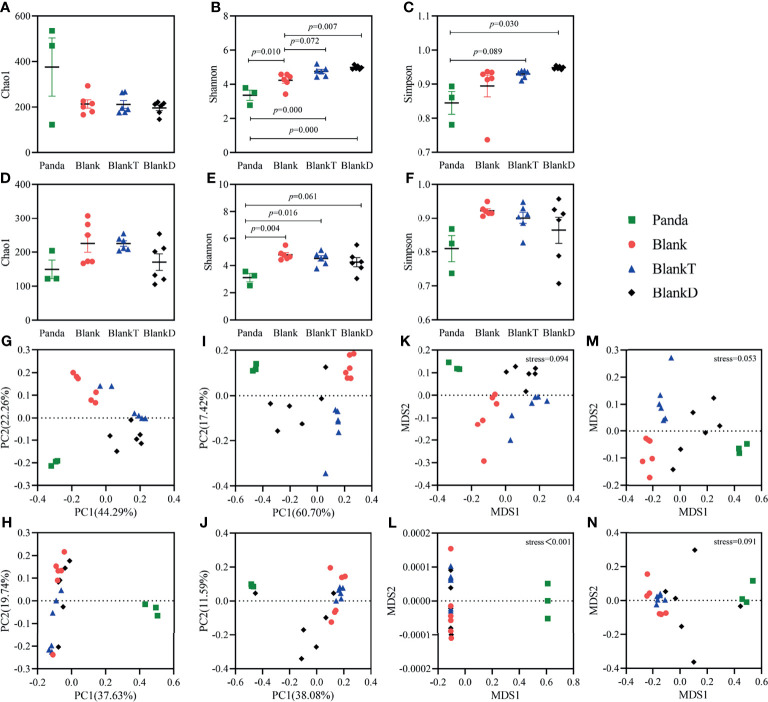
Comparison of intestinal microbiota diversity among Panda, Blank, BlankT and BlankD group on days 13 and 16. Results of alpha diversity analysis on day 13 **(A–C)** and day 16 **(D–F)**, the data are shown as mean ± SEM. Blank, n = 6; Panda, n = 3; BlankT, n = 6. Data were analyzed with one-way ANOVA (Tukey’s test). Principal coordinate analysis (PCoA) **(G, H)** and non-metric multidimensional scaling (NMDS) **(K–L)** were performed using unweighted UniFrac **(G, K)** and weighted UniFrac **(H, L)** distances on day 13. PCoA **(I, J)** and NMDS **(M, N)** analyses were performed using unweighted UniFrac **(I, M)** and weighted UniFrac **(J, N)** distances on day 16.

Panda-microbiota associated (PMA) mice were generated by pre-treatment with an antibiotic cocktail for 7 days and then gavage administration of panda microbiota for 3 days. We continuously monitored total bacteria, *Bifidobacteria*, *Enterococcus*, *Escherichia coli*, and *Lactobacillus* by viable bacterial count from days 0 to 13. Fecal microbiota were mostly eliminated by day 7 of the antibiotic cocktail (**Supplementary Table 2**). By day 13, there were no differences observed in the absolute counts of detected bacteria aside from the higher total anaerobe count in the Blank and BlankT group (**Supplementary Table 2**). To assess for similarity of microbiome diversity and composition of the PMA mice with the donor panda microbiota, 16S rRNA sequencing was performed of the panda gut microbiome (Panda group), non-transplanted mice (Blank group), and PMA-mice (BlankT group). Chao1, Shannon, and Simpson indices were measured to assess the alpha diversity of the gut microbiome (**Figure 1**). On day 13 (**Figures 1A**–**C**), the Shannon index was the only alpha diversity metric with a significant difference. As shown in the figure, the Shannon index in the Blank and BlankT groups was higher than that of the Panda group, while no difference was observed between the Blank and BlankT groups. More differences in alpha diversity were observed on day 16 (**Figures 1D**–**F**): the Chao1, Shannon, and Simpson indices were higher in the BlankT group compared to the Panda group, but only the Shannon index showed a significant difference. There continued to be no differences observed between the Blank and BlankT groups. The BlankT group had lower abundance of *Enterococcus*, *Lactobacillus* and *Parasutterella* than the Blank group and higher abundance of *Enterobacteriacaeae* (including *Klebsiella*, *Morganella* and unclassified members of this family) at day 13 by DESeq2 analysis (**Supplementary Figures 2A, B** and **Supplementary Tables 3A, B**) on day 13. Data from day 16 showed a similar result as that on day 13, including increased *Enterobacteriacaeae* in the BlankT group (*Klebsiella*, *Providencia*, *Proteus*, and unclassified members) (**Supplementary Figures 2C, D** and **Supplementary Tables 3C, D**). The PCoA and NMDS results (**Figures 1G**–**N**) indicated that the gut microbiome of the BlankT group separated from the Panda group and the Blank group, which was statistically significant based on Anosim and Adonis analysis (**Supplementary Table 4**) on both days 13 and 16. These results suggest that the gut microbiome of the PMA-mouse is different from the non-transplanted mice and that this difference may persist.

The DSS-induced colitis mouse model has similar clinical features to pandas with a high abundance of *Enterobacteriaceae* and inflammation caused by an epithelial barrier disruption with exposure of mucosal immune cells to microbial antigens. DSS (5%) was supplied in the drinking water for 3 days following panda microbiota administration to generate a DPMA mouse to model intestinal inflammation in pandas. After DSS oral administration (on day 13), the BlankT and BlankD groups had higher alpha diversity than the Panda group (**Figures 1A**–**C**). Beta diversity results (**Figures 1G–N**) showed that the different groups separated from each other, which was significant by Anosim and Adonis analyses (**Supplementary Table 5**). Furthermore, taxonomic summary plots at the family level showed that the proportion of *Enterobacteriaceae* and *Enterococcaceae* was higher in the BlankD and Panda groups than the BlankT group (**Supplementary Figures 3A, B**). DESeq2 analysis at the genus level similarly demonstrated enrichment of *Enterococcus* and unclassified *Enterobacteriaceae* (**Supplementary Table 6**). On day 16, the panda group had lower alpha diversity by the Shannon index compared to the BlankT group but there were otherwise no significant differences across the three groups (**Figures 1D**–**F**). The PCoA and NMDS (**Figures 1K**–**N**) results indicated that there were significant differences among groups based on Anosim and Adonis analyses (**Supplementary Table 6**) on days 13 and 16. At the family level, *Enterobacteriaceae* and *Enterococcaceae* were found in much greater abundance in the BlankD and Panda groups compared to the BlankT group (**Supplementary Figures 3C, D**). Viable bacterial counts of *E. coli* (a member of the *Enterobacteriacaeae* family) were significantly greater in BlankD compared to BlankT; both groups had higher *E. coli* and *Enterococcus* as well as lower *Bifidobacterium* than panda microbiome (**Supplementary Table 7**).

To identify genera affected by intestinal inflammation in the DPMA-mouse, we further compared the intestinal microbiota between PMA-mouse (BlankT group) and DPMA-mouse (BlankD group). On day 13, LefSe analysis (**Supplementary Figure 4A**) indicated that, at the genus level, the abundance of *Enterorhabdus*, *Bifidobacterium*, *Roseburia*, *Dubosiella*, *Faecalibaculum*, and *Akkermansia* were decreased, and the abundance of *Bacteroides*, *Enterococcus*, *Clostridioides*, *Romboutsia*, *Clostridium_methylpentosum*, *Turicibacter*, *Veillonella*, *Klebsiella*, *Proteus*, *Providencia*, *Pseudomonas*, and *Parasutterella* were increased after DSS administration. On day 16, LefSe analysis (**Supplementary Figure 4B**) revealed that the abundance of *Enterorhabdus*, *Alistipes*, *Roseburia*, *Butyricicoccus*, *Caproiciproducens*, *Dubosiella*, *Faecalibaculum*, *Anaeroplasma*, and *Akkermansia* were decreased, and the abundance of *Enterococcus*, *Clostridioides*, *Paeniclostridium*, *Romboutsia*, *Flavonifractor*, *Veillonella*, *Klebsiella*, *Lelliottia*, *Morganella*, *Proteus*, and *Providencia* were increased after DSS administration. As differential genera varied between days 13 and 16, we decided to utilize genera with the same change trend on both days 13 and 16 as marker genera for intestinal inflammation. This included *Enterorhabdus* spp., *Roseburia* spp., *Dubosiella* spp., *Faecalibaculum* spp., *Akkermansia* spp., *Enterococcus* spp., *Clostridioides* spp., *Romboutsia* spp., *Veillonella* spp., *Klebsiella*, spp. *Proteus* spp. and *Providencia* spp.

### *L. plantarum* G83 Ameliorates DSS-Induced Disruption of the Intestinal Barrier

**Figure 2:**
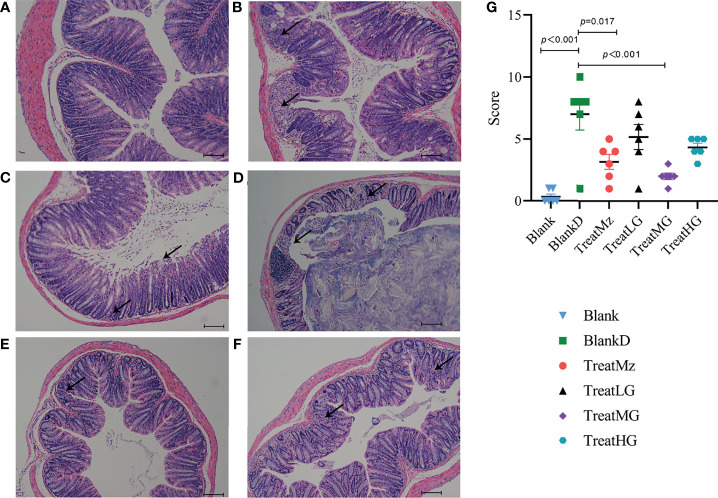
Representative images and histopathology score of the colon of DPMA mice (40× magnification). Colonic epithelial structure was destroyed, and a large amount of inflammatory cell infiltration was observed in the BlankD group **(B)** compared to the blank group **(A)**, while those traits were alleviated in the TreatMz group **(C)**. There were mild epithelial changes and inflammatory cell infiltration observed in the TreatLG **(D)**, TreatMG **(E)**, and TreatHG **(F)** groups. **(G)** Histological scores (0–12 scale) for each group. All data are shown as mean ± SEM and analyzed with one-way ANOVA (Tukey’s test).

**Figure 3:**
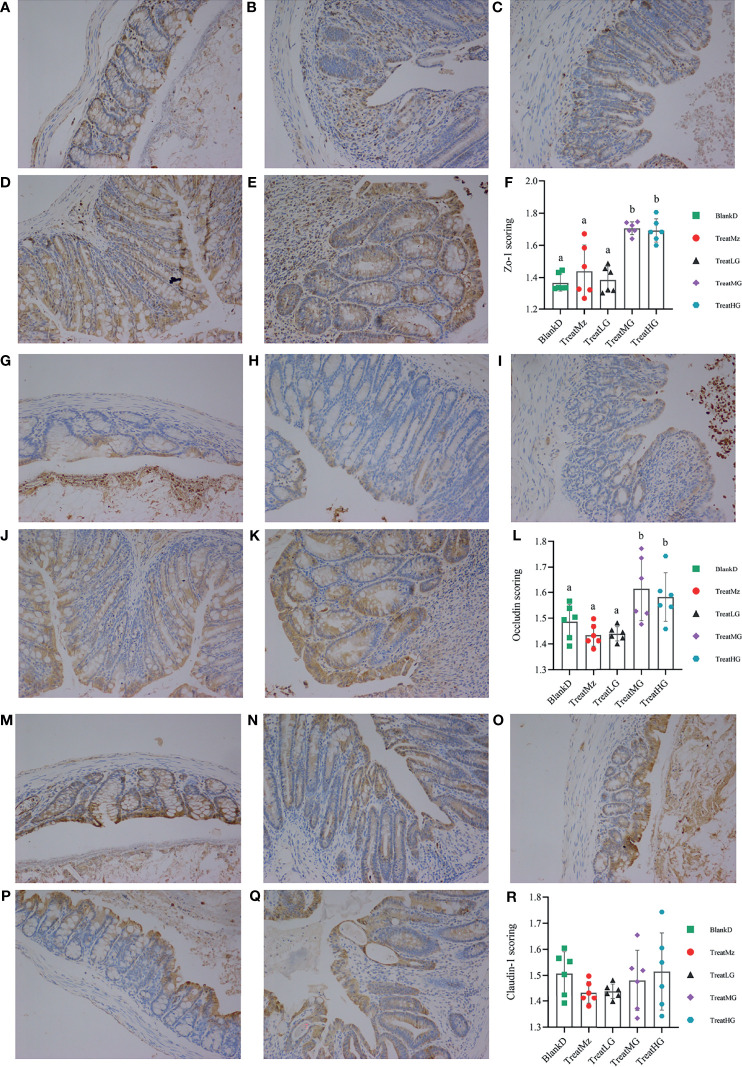
Representative images and scoring results of Tj proteins in the colon of DPMA mice, as detected by immunohistochemistry (200× magnification). There was no significant difference among the BlankD **(A)**, TreatMz **(B)** and TreatLG **(C)** groups. The expression of ZO-1 in TreatMG **(D)** and TreatHG **(E)** groups was significantly higher than in other groups; ZO-1 scores are shown in panel **(F)**. There was no significant difference among the BlankD **(G)**, TreatMz **(H)**, and TreatLG **(I)** groups. The expression of Occludin in TreatMG **(J)** and TreatHG **(K)** groups was significantly higher than in other groups; scores are shown in panel **(L)**. There was no significant difference in the expression of Claudin-1 protein among groups **(M)**: BlankD, **(N)**TreatMz, **(O)** TreatLG, **(P)** TreatLG, **(Q)** TreatHG), but the scores **(R)** showed a trend for increased expression of claudin-1 protein in the TreatMG and TreatHG groups. All data are shown as mean ± SEM and analyzed with one-way ANOVA (Tukey’s test). Significance is denoted by letters; if two groups have different letters, this indicates that the difference between these groups was significant with p <0.05.

**Figure 4:**
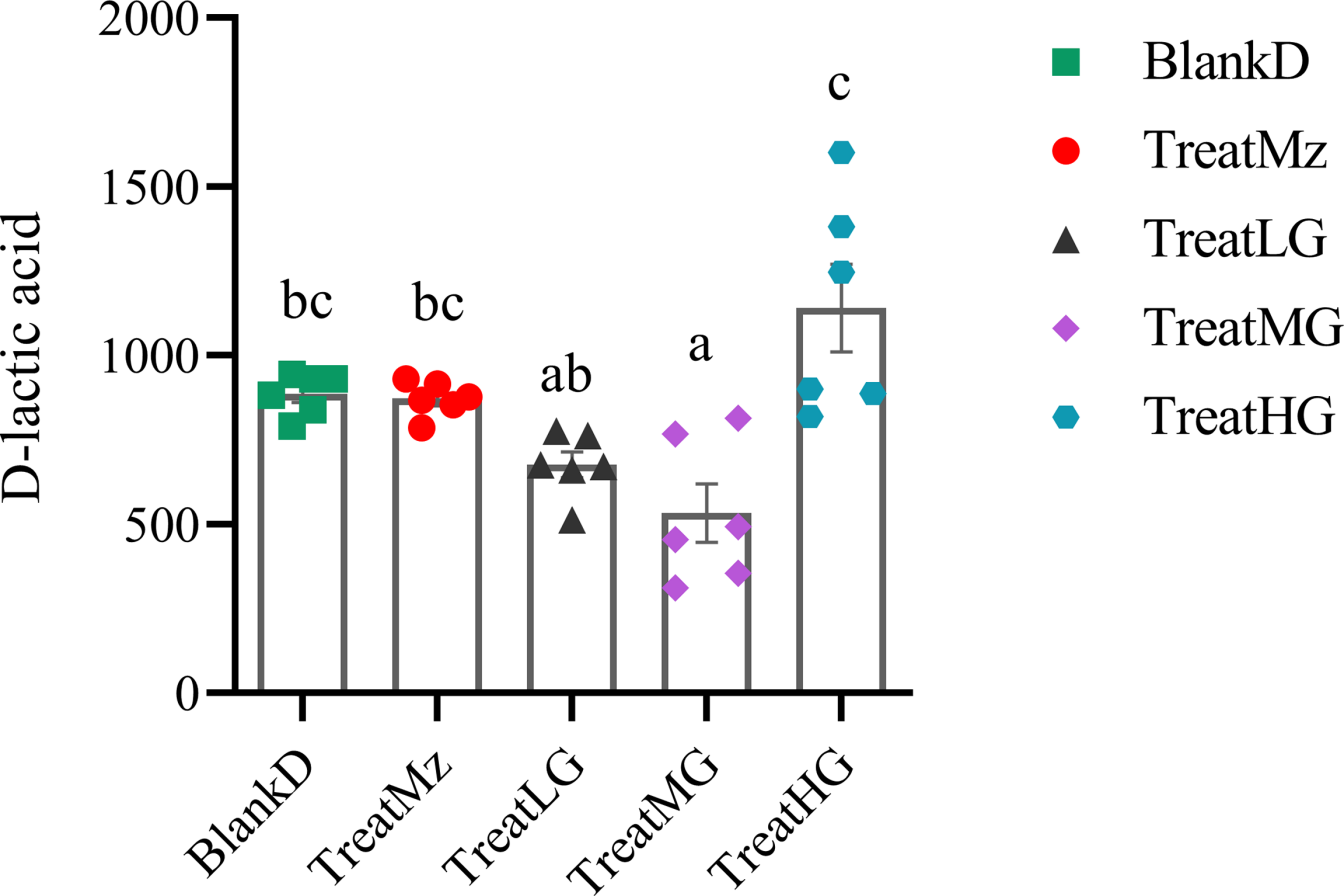
*L. plantarum* G83 decreases concentration of D-lactic acid in the serum of DPMA mice. D-lactic acid concentration in the serum was assessed by colorimetric method. Lower D-lactic acid was observed in the TreatLG and TreatMG groups which was significant only for TreatMG relative to BlankD. All data are shown as mean ± SEM. Data were analyzed with one-way ANOVA (Tukey’s test). Significance is denoted by letters; if two groups have different letters, this indicates that the difference between these groups was significant with p <0.05.

Based on prior reports that *Lactobacillus* strains can enhance intestinal barrier function, we investigated whether the panda-derived *L. plantarum* G83 strain would have this effect on the DPMA-mouse model. Three different doses of *L. plantarum* G83 were provided to DPMA mice after administration of 5% DSS drinking water for 3 days (TreatLG, TreatMG, and TreatHG as described in the *Material and Methods*). As a treatment positive control, we also included a group receiving mesalazine, an anti-inflammatory medication that ameliorates DSS-induced colitis (34). First, we performed a histopathologic analysis of the colon tissue (**Figure 2**). Results showed that the epithelial cell integrity of the colon tissue was destroyed by DSS administration, and a large amount of inflammatory cell infiltration was observed in the BlankD group. There was, however, significantly reduced ulcer damage, intestinal epithelial disruption, and inflammatory cell infiltration by histopathology scoring in the mesalamine (TreatMz) and medium dose *L. plantarum* G83 treatment group (TreatMG) compared with the BlankD group. We next investigated the effect of *L. plantarum* G83 on tight junction proteins in the DPMA mouse. The expression of ZO-1 and Occludin protein but not Claudin-1 were significantly increased in the TreatMG and TreatHG groups by immunohistochemistry (**Figure 3**). To further assess intestinal permeability, the concentration of D-lactic acid in the serum was measured (**Figure 4**). Compared to the BlankD group, only the TreatMG group had a significantly lower concentration of D-lactic acid. Altogether, data from these experiments support that *L. plantarum* G83 can modulate epithelial barrier integrity in the DPMA mouse.

### *L. plantarum* G83 Ameliorates Colitis in DPMA Mice

**Figure 5:**
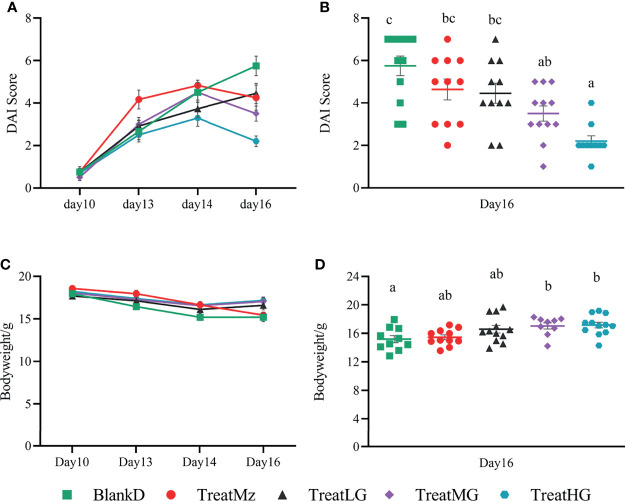
*L. plantarum* G83 improves DAI score and body weight loss in DPMA mice. DSS induced colitis symptoms including weight loss, diarrhea, and rectal bleeding that were monitored by DAI score **(A)**. TreatMG and TreatHG groups showed a lower DAI score on day 16 **(B)**, while other groups had no difference compared with BlankD group. *L. plantarum* G83 treated mice also exhibited less weight loss than the mice in BlankD and TreatMz groups **(C, D)**. All data are shown as mean ± SEM. Data were analyzed with one-way ANOVA (Tukey’s test). Significance is denoted by letters; if two groups have different letters, this indicates that the difference between these groups was significant with p <0.05.

**Figure 6:**
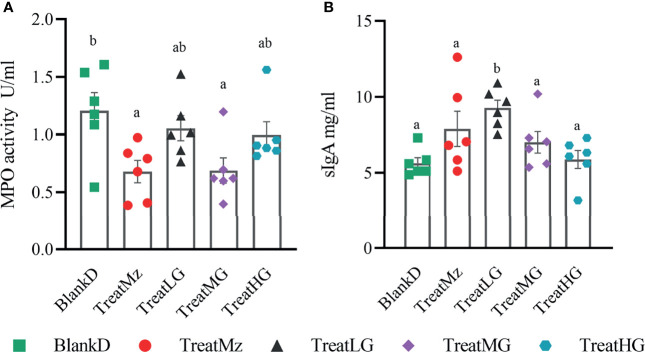
Concentration of sIgA and MPO activity in the colon of DPMA mice. MPO activity **(A)** was decreased in all treatment groups, but only TreatMz and TreatMG groups showed significant differences. sIgA **(B)** was only increased in the TreatLG group, other groups had no difference compared with BlankD group. All data are shown as mean ± SEM. Data were analyzed with one-way ANOVA (Tukey’s test). Significance is denoted by letters; if two groups have different letters, this indicates that the difference between these groups was significant with p <0.05.

**Figure 7:**
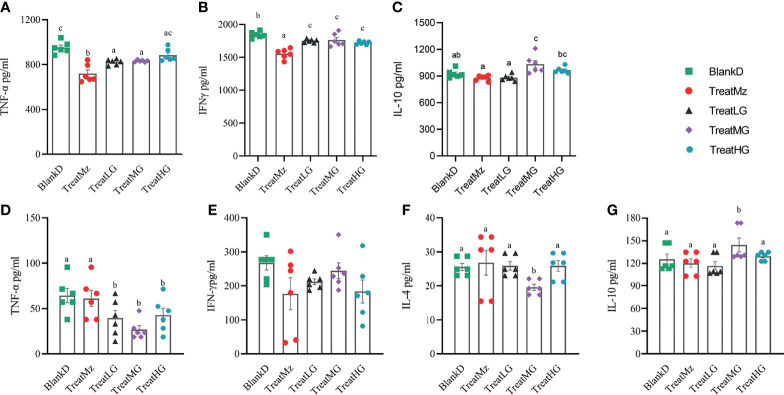
*L. plantarum* decreases pro-inflammatory cytokines in the serum and colon tissue of DPMA mice. In the serum, TNF-α **(A)** and IFN-γ **(B)** were significantly inhibited by *L.plantarum* G83 administration but less than the mesalazine treated group. IL-10 **(C)** was increased in the TreatMG and TreatHG groups, but not in the TreatMz and TreatLG groups. In the colon tissue, TNF-α **(D)** decreased after *L. plantarum* G83 treatment, while there was no significant difference observed in IFN-γ **(E)** between groups. IL-4 **(F)** was decreased only in the TreatMG group, other groups had no difference compared with the BlankD group. IL-10 **(G)** was increased only in the TreatMG group; other groups had no difference compared with the BlankD group. All data are shown as mean ± SEM. Data were analyzed with one-way ANOVA (Tukey’s test). Significance is denoted by letters; if two groups have different letters, this indicates that the difference between these groups was significant with p <0.05.

DSS induced colitis symptoms in the DPMA mice, namely, weight loss, diarrhea, and rectal bleeding were monitored by a disease activity index (DAI). Reduced DAI scores were observed at day 16 in the DPMA mice receiving *L. plantarum* G83, which was significant for the TreatMG and TreatHG groups (**Figures 5A, B**). *L. plantarum* G83 treated mice also exhibited less weight loss than the mice in the BlankD group which again was significant only for the TreatMG and TreatHG groups (**Figures 5C, D**). Colon myeloperoxidase (MPO) activity—a marker of colonic inflammation—was significantly reduced in the TreatMz and TreatMG groups compared to BlankD (**Figure 6A**). Based on these results, we aimed to determine whether *L. plantarum* G83 could reduce serum and colonic levels of inflammatory cytokines in the DPMA mice. Proinflammatory cytokines in the serum, including TNF-α and IFN-γ, were significantly inhibited by *L. plantarum* G83 administration but less than the mesalazine treated group (**Figures 7A, B**). IL-10, which has anti-inflammatory effects, was promoted by *L. plantarum* G83 treatment in the medium high-dose group (**Figure 7C**). We did not observe any difference in IL-10 between the other three groups and BlankD. Colonic TNF-α was inhibited by *L. plantarum* G83 administration at all three doses while unexpectedly, mesalazine failed to reduce TNF-α secretion in the colon tissue (**Figure 7D**). IFN-γ levels in colon tissue did not significantly differ among the groups (**Figure 7E**). All treatment groups failed to promote colonic IL-10 except for the medium dose of the *L. plantarum* G83, which showed a higher concentration of IL-10 (**Figure 7G**). Conversely, the TreatMG group exhibited a lower colonic IL-4 concentration compared to the BlankD group, and all other groups showed no difference (**Figure 7F**). Secretory immunoglobulin A (sIgA) in luminal content increased only in the TreatLG group, and there were no significant differences among the other groups (**Figure 6B**). Overall, these results demonstrated that *L. plantarum* G83 exhibits an anti-inflammatory effect in the DPMA mice.

### *L. plantarum* G83 Improves Dysbiosis in DPMA Mice

**Figure 8:**
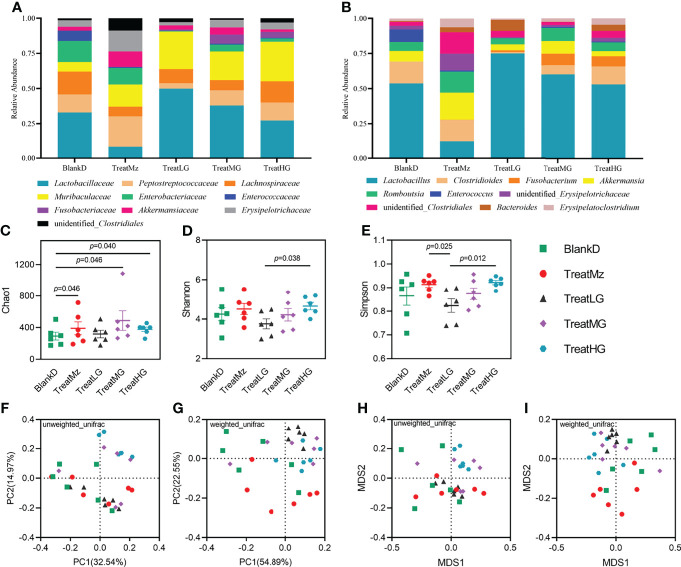
*L. plantarum* G83 treatment improves microbiota diversity in DPMA mice. Relative abundance of the microbiota at the family level **(A)**. The relative abundances of *Peptostreptococcaceae*, *Enterobacteriaceae*, and *Enterococcaceae* were lower in all *L. plantarum* G83 treatment groups compared with BlankD. The relative abundances of *Muribaculaceae* and *Fusobacteriaceae* in all treatment groups were higher than that in BlankD group. Higher relative abundance of *Lactobacillaceae* was observed in the TreatLG group. At the genus level **(B)**, the TreatLG group had the highest relative abundance of *Lactobacillus* in all groups. There was a higher relative abundance of *Fusobacterium* in *L. plantarum* G83 treatment groups. The relative abundance of *Enterococcus* in all *L. plantarum* G83 treatment groups was lower than that in BlankD group. However, the intestinal microbiota structure of the TreatMz group was different from that of other groups; at the genus level, it had a lower relative abundance of *Lactobacillus*, *Fusobacterium*, and *Enterococcus* but a higher relative abundance of *Akkermansia*. *L. plantarum* G83 treatment increased the Chao1 index in the DPMA mice **(C)**. The Shannon index and Simpson index were significantly higher in the TreatHG group than in the TreatLG group **(D, E)**. PCoA **(F, G)** and NMDS **(H, I)** analysis showed that the intestinal microbiota of all treatment groups differed from each other based on Anosim and Adonis tests (**Supplementary Table S9**).

We hypothesized that the beneficial effects of *L. plantarum* G83 on intestinal inflammation and barrier integrity in DPMA mice were due to reduced dysbiosis. To assess this, we compared the colonic luminal microbiota of the DPMA mouse after receiving the different treatments. As shown in **Figure 8**, *L. plantarum* G83 treated groups had a lower abundance of *Peptostreptococcaceae*, *Enterobacteriaceae*, and *Enterococcaceae*, and a higher abundance of *Muribaculaceae* and *Fusobacteriaceae* compared to the BlankD group. Higher relative abundance of *Lactobacillaceae* was observed in all *L. plantarum* G83 treatment groups compared to the TreatMz group (**Figure 8A**). At the genus level, the relative abundance of *Fusobacterium* increased in all three G83 treatment groups, and the relative abundance of *Lactobacillus* increased in all *L. plantarum* G83 treatment groups compared to the TreatMz group (**Figure 8B**). DESeq2 analysis showed that *Fusobacterium* enrichment and *Enterococcus* depletion were statistically significant in all three G83 dose groups (**Supplementary Table 8**). When we analyzed the gut microbiota of the TreatMz group, we found that the microbiota of the TreatMz group showed a drastic change, different from any of all other groups. The relative abundance of *Lactobacillus* was greatly reduced compared to other groups. The TreatMz, TreatLG, and TreatHG groups had increased microbial richness compared with the BlankD group by the Chao1 index (**Figure 8C**). The treatment groups did not show statistically significant differences in Shannon index or Simpson index compared to BlankD (**Figures 8D, E**). Beta diversity analysis (**Figures 8F, G**) showed that there was a significant difference between each *L. plantarum* G83 treated group and the BlankD or TreatMz group; no significant difference was observed between the BlankD and TreatMz groups based on Anosim and Adonis analyses (**Supplementary Table 9**). To understand the effect of *L. plantarum* G83 on DSS-induced microbial shifts, we performed LefSe analysis to analyze changes in the DSS biomarker genera in all five groups. *Enterococcus* spp., *Klebsiella* spp., *Proteus* spp. and *Providencia* spp. were more abundant in both the BlankD and TreatMz groups (**Supplementary Figure 5**). These genera belong to the family *Enterobacteriaceae*, typically considered as a marker of high risk for enteritis in pandas. *Veillonella* spp. only increased in the BlankD group. Besides these changes in the previously defined DSS biomarker genera, LefSe also demonstrated enrichment of *Bifidobacterium* spp. in the Treat MG and TreatHG groups (**Supplementary Figure 5F**). Q-PCR quantitation and viable bacterial count were performed to determine changes in absolute abundance of specific microbial taxa. All three *L. plantarum* G83 treatment groups had reduced *Enterobacteraceae* by q-PCR compared to the BlankD and TreatMz groups, and TreatLG and TreatMG also had reduced Proteobacteria (**Supplementary Figure 6**). Lower abundance of *E. coli* was detected in all three *L. plantarum* G83 treatment groups compared to the BlankD group (**Supplementary Figure 7**). The treatMG and TreatHG groups also had greater *Bifidobacterium* spp., consistent with the 16S sequencing analysis result. FAPROTAX was used to predict the function of the bacteria in the intestinal microbiota. The heatmap result showed that the BlankD group had a high abundance of genes associated with human_pathogen_diarrhea, human_pathogen_all, human_pathogen_pneumonia, human_pathogen_septicemia, ureolysis, and nitrate metabolism, while the mesalazine and *L. plantarum* G83 treatment groups had a very low abundance of genes in these pathways (**Supplementary Figure 8**). Overall, *L. plantarum* G83 improved microbial dysbiosis, with decreased levels of *Enterobacteriaceae* and disease-related genes.

### *L. plantarum* G83 Increases Luminal Short-Chain Fatty Acid (SCFA) Concentrations in DPMA Mice

**Figure 9:**
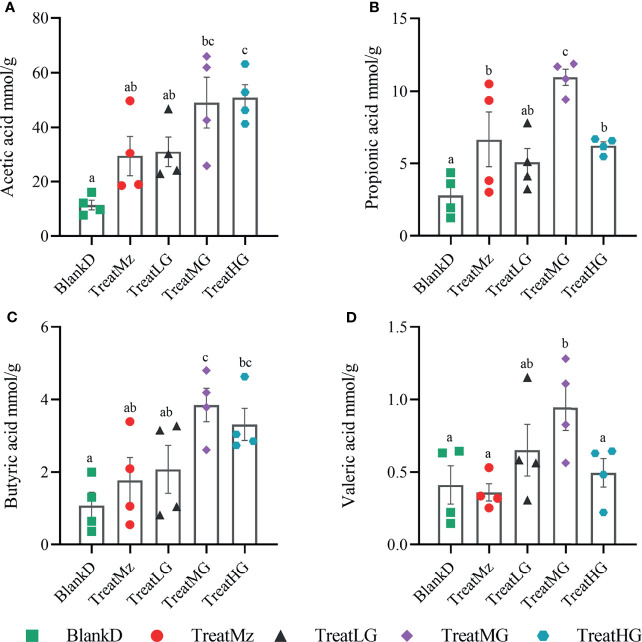
*L. plantarum* G83 increases luminal short-chain fatty acid (SCFA) concentration in DPMA mice. SCFAs—including acetic acid **(A)**, propionic acid **(B)**, butyric acid **(C)**, and valeric acid (valeric acid and isovaleric acid) **(D)**—in the colon lumen were measured by high-performance gas chromatography (HPGC). The medium dose of *L. plantarum* G83 increased luminal concentration of all detected SCFAs compared to the other groups. The TreatMz and the TreatLG groups had numerically increased SCFA concentrations compared to the BlankD group, but only propionic acid **(B)** in the TreatLG group showed significance. All data are shown as mean ± SEM, n = 4. Data were analyzed with one-way ANOVA (Tukey’s test). Significance is denoted by letters; if two groups have different letters, this indicates that the difference between these groups was significant with p <0.05.

To further investigate the functional consequences of the altered microbiome induced by *L. plantarum* G83 treatment, we measured the concentration of SCFAs, including acetic acid, propionic acid, butyric acid, and valeric acid (valeric acid and isovaleric acid) in the colon lumen using high-performance gas chromatography (HPGC). The medium dose of *L. plantarum* G83 increased luminal concentration of all detected SCFAs including acetic acid, propionic acid, butyric acid, and valeric acid compared to the BlankD, TreatMz, and TreatLG groups (**Figure 9**). The high dose of G83 had similar concentrations of acetic acid and butyric acid as the medium dose, but lower concentrations of propionic acid and valeric acid. Furthermore, the TreatMz and the TreatLG groups had numerically increased SCFA concentrations compared to the BlankD group, but only propionic acid in the TreatLG group showed significance. Together, the medium and high dose of *L. plantarum* G83 administration showed a strong capability to increase colonic luminal SCFAs.

### *L. plantarum* G83 Inhibits NF-κB/p65 Expression in the Colon of DPMA Mice

**Figure 10:**
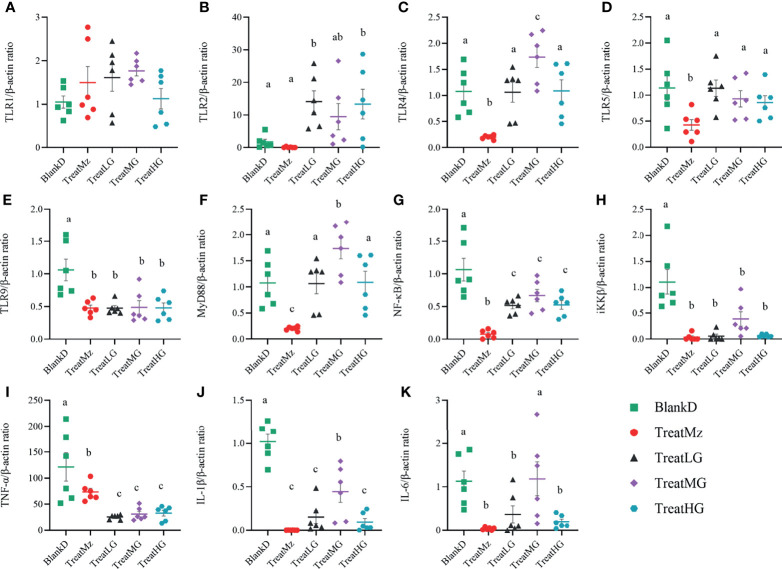
Expression of TLRs and NF-κB relevant genes in DMPA mice. Gene expression in colonic tissue was determined by q-PCR. All treatment groups had no significant effect on TLR1 gene expression **(A)**. TLR2 was activated in all *L. plantarum* G83 treatment groups **(B)**. The TreatMz group had reduced TLRs, including TLR4 **(C)** and TLR5 **(D)**. TLR9 **(E)** was decreased in all treatment groups. MyD88 **(F)** and NF-κB **(G)** was increased by *L. plantarum* G83 treatment compared with mesalazine treatment. IKKβ **(H)**, TNF-α **(I)**, IL-1β **(J)** and IL-6 **(K)** were downregulated in all treatment groups compared with BlankD group. All data are shown as mean ± SD, n = 6. Data were analyzed with one-way ANOVA (Tukey’s test). Significance is denoted by letters; if two groups have different letters, this indicates that the difference between these groups was significant with p <0.05.

We hypothesized that alterations in the microbiome and microbial metabolites including SCFAs would affect intestinal immune cell signaling pathways including the NF-κB/p65 pathway that regulate the inflammatory response. To investigate this, we compared colonic tissue expression of Toll-like receptors (TLRs) and the NF-κB/p65 pathway related genes among the groups by q-PCR. *L. plantarum* G83 groups had increased TLR2 gene expression, whereas mesalazine did not alter TLR2 gene expression (**Figures 10A**–**E**). The expression level of the TLR4 and TLR5 genes was significantly decreased by mesalazine treatment. We did not observe any effects of *L. plantarum* G83 on TLR4 and TLR5 gene expression, except that the medium dose of *L. plantarum* G83 showed increased TLR4 gene expression. Interestingly, TLR9, the receptor for bacterial CpG-DNA, was inhibited by both mesalazine and *L. plantarum* G83. There was no difference among groups in TLR1 expression.

To confirm the effect of *L. plantarum* G83 on the inflammatory response, we measured expression levels of genes related to the NF-κB pathway, which is critically important in the immune response. Mesalazine significantly reduced the gene expression level of MyD88, NF-κB, iKKβ, TNF-α, IL-1β, and IL-6 (**Figures 10F–K**). The *L. plantarum* G83 treated group also showed reduced expression of NF-κB, iKKβ, TNF-α, IL-1β, and IL-6. Aside from the medium dose, *L. plantarum* G83 treatment did not affect MyD88 gene expression.

**Figure 11:**
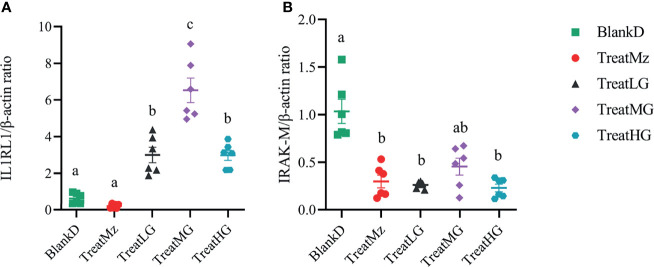
*L. plantarum* G83 treatment was associated with altered gene expression in colon of TLR receptor negative regulators. IL1R1 **(A)** expression was increased in all *L. plantarum* G83 groups with the highest gene expression level observed in the medium dose treatment group. All treatment groups had reduced IRAK-M **(B)** gene expression compared to BlankD. All data are shown as mean ± SEM, n = 6. Data were analyzed with one-way ANOVA (Tukey’s test). Significance is denoted by letters; if two groups have different letters, this indicates that the difference between these groups was significant with p <0.05.

We noted that there was an increased TLR expression despite a reduction in the MyD88 expression. To investigate this, we further tested several TLR negative regulators, including interleukin 1 receptor-like 1 receptor (IL1RL1) and IL-1R-associated kinase M (IRAK-M). The IL1RL1 gene was promoted by *L. plantarum* G83 with the highest gene expression level observed in the medium dose treatment group. All treatment groups downregulated IRAK-M gene expression (**Figure 11**).

**Figure 12:**
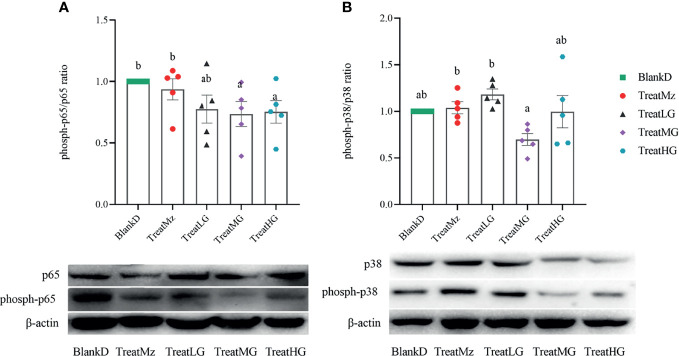
*L. plantarum* G83 increases dephosphorylation of NF-κB-p65. NF-κB-p65 **(A)** and MAPK-p38 **(B)** phosphorylation status was assessed by Western blot. Reduced ratio of phospho-p65/p65 was seen in all three *L. plantarum* G83 treatment groups, which was significant for TreatMG and TreatHG. There were no significant group differences in p-MAPK-p38 compared to BlankD. All data are shown as mean ± SEM, n = 5. Data were analyzed with one-way ANOVA (Tukey’s test). Significance is denoted by letters; if two groups have different letters, this indicates that the difference between these groups was significant with p <0.05.

We further investigated NF-κB activity by assessing phosphorylation status by Western blot for p-NF-κB-p65/NF-κB-p65 and p-MAPK-p38/MAPK-p38. In the present study, *L. plantarum* G83 treatment led to reduced NF-κB-p65 phosphorylation which was significant for the TreatMG and TreatHG groups. However, p-MAPK-p38 did not significantly differ in any of the groups compared to BlankD (**Figure 12**). In summary, these data indicated that the *L. plantarum* G83 inhibited the NF-κB/p65 pathway in the colon of DPMA mice.

### *L. plantarum* G83 Altered Th1/Th2 and Th1/Th17 Balance in DPMA Mice

**Figure 13:**
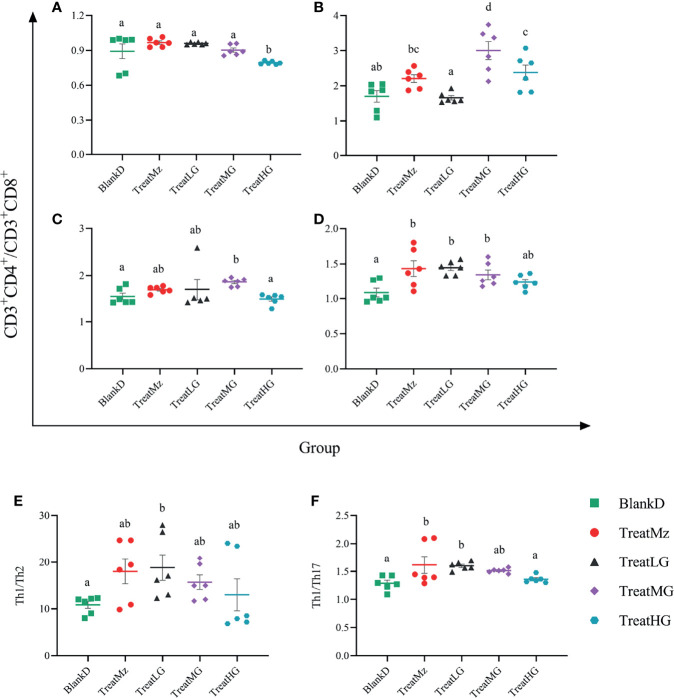
*L. plantarum* G83 treatment altered Th1/Th2 and Th1/Th17 balance in DPMA mice. The ratio of CD3+CD4+/CD3+CD8+ T cells was measured by flow cytometry in peripheral blood **(A)**, Peyer’s patches **(B)**, mesenteric lymph nodes (MLN) **(C)**, and spleen **(D)**. In peripheral blood, the CD3^+^CD4^+^/CD3^+^CD8^+^ ratio was reduced in the TreatHG group compared to the other groups. In Peyer’s patches **(B)**, the CD3^+^CD4^+^/CD3^+^CD8^+^ ratio was elevated in the TreatMG and TreatHG groups compared to the other groups. However, CD3^+^CD4^+^/CD3^+^CD8^+^ T cells in the MLN were only promoted in the TreatMG group **(C)**. Also, the CD3^+^CD4^+^/CD3^+^CD8^+^ T cells in the spleen were improved by the mesalazine, low and medium dose of *L. plantarum* G83 **(D)**. Th1/Th2 **(E)** cell and Th1/Th17 **(F)** cell were elevated by mesalazine, low and medium dose of the *L. plantarum* G83 treatments; the mesalazine and low dose *L. plantarum* G83 groups had the highest ratio. All data are shown as mean ± SEM, n = 6. Data were analyzed with one-way ANOVA (Tukey’s test). Significance is denoted by letters; if two groups have different letters, this indicates that the difference between these groups was significant with p <0.05.

Flow cytometry was then performed to determine the polarization of different T cell subsets in the peripheral blood, Peyer’s Patches, mesenteric lymph nodes (MLN), and spleen. In the peripheral blood, the ratio of CD3^+^CD4^+^/CD3^+^CD8^+^ T cells was reduced by high dose *L. plantarum* G83 compared to the other groups (**Figure 13A**). In contrast, the CD3^+^CD4^+^/CD3^+^CD8^+^ ratio in spleen was increased in the mesalazine, low and medium dose *L. plantarum* G83 groups compared to untreated DPMA mice (**Figure 13D**). The CD3^+^CD4^+^/CD3^+^CD8^+^ ratio was also elevated in Peyer’s patches in the medium and high dose *L. plantarum* G83 groups compared to the other groups (**Figure 13B**). The medium dose *L. plantarum* G83 group also had increased CD3^+^CD4^+^ T/CD3^+^CD8^+^ ratio in the mesenteric lymph nodes compared to BlankD (**Figure 13C**). We then assessed T helper cell (CD4^+^) subsets in the spleen including Th1, Th2, and Th17 cells. We found that the ratio of Th1/Th2 cells was increased in the low dose *L. plantarum* G83 group compared to BlankD (**Figure 13E**). The low dose *L. plantarum* G83 group and the mesalazine group also had higher Th1/Th17 ratio compared to BlankD (**Figure 13F**). Taken together, these results suggest that *L. plantarum* G83 treatment could affect T cell polarization in DPMA mice.

## Discussion

Gastrointestinal disease is reported to be a main factor affecting the health of captive pandas (35). One of the reasons is because the intestinal barrier of the giant panda is disturbed by indigestible bamboo and parasites. Due to the high abundance of *Enterobacteriaceae* in their intestines, pandas often suffer intestinal infection and inflammation. Moreover, long-term captivity and antibiotic misuse further promote dysbiosis, increasing susceptibility to pathogenic bacteria in the intestines of the giant panda (36, 37). To study intestinal inflammation related to the panda microbiome, we developed panda microbiota-associated mice, generated by antibiotic treatment followed by oral gavage with panda fecal microbiota. These mice showed significant differences in microbial composition compared to control antibiotic-treated mice but also the donor panda microbiota. PMA mice had expanded *Enterobacteriacaeae* populations compared to antibiotic-treated controls, supporting that transferred giant panda intestinal microbes had modulated the microbiome of PMA mice and potentially successfully colonized though 16S rRNA gene sequencing did not provide sufficient resolution to confirm the presence of donor-derived bacterial strains. The persistent difference between PMA mice and the panda donor microbiota reflects the challenges of microbiota transfer, which are apparent even when transferring across hosts of the same species and greatly exacerbated when transferring microbes across distinct mammalian hosts (30).

DSS was introduced to induce intestinal inflammation in panda microbiota-associated mice. DSS-induced colitis is a result of the damage to the epithelial monolayer, which allows microbes and their antigens to infiltrate into underlying tissues, triggering an intestinal immune response (38). In giant pandas, similar intestinal inflammation by opportunistic pathogens often occurs when the epithelium is damaged by dietary habits and parasitic infections. DSS-treated PMA mice showed a higher abundance of *Enterobacteriaceae* compared to the BlankT group, which is consistent with the high abundance of these microbes in giant pandas with intestinal inflammation. These findings support that DSS-induced giant panda microbiota-associated mice are a pertinent model for studying giant panda intestinal inflammation.

The DSS-induced PMA model was used to assess the effect of administration of *L. plantarum* G83 on intestinal inflammation. In this study, mice in the BlankD group developed bloody diarrhea and weight loss from days 13 to 16. *L. plantarum* G83 treatment at the medium and high doses mitigated body weight loss and DAI score, reflecting amelioration of colitis (39). Decreased severity of clinical parameters also corresponded to reduced histologic disease severity in the medium dose group and improvement in cytokine profiles, namely, reduced serum and colonic TNF-α and reduced serum IFN-γ. Furthermore, IL-10, a vital anti-inflammatory cytokine in improving the host immune response against pathogen invasion and preventing inflammatory diseases (40), was significantly increased in the medium dose of the *L. plantarum* G83 treatment group compared with other treatment groups.

Amelioration of intestinal inflammation by *L. plantarum* G83 supplementation may reflect beneficial effects on intestinal barrier function. TJ protein serves as a gatekeeper for the paracellular pathway to preserve tissue homeostasis (41). Thus, macromolecular substances from the intestinal lumina may easily enter the host through the paracellular pathway when the epithelial cell permeability is challenged by an inflammation. Many studies have shown that *Lactobacillus* can decrease paracellular permeability in LPS- or pathogen-impaired Caco-2 monolayer cells, as determined by FITC-D4 (11, 42). Resta-Lenert reported that *L. acidophilus* ATCC4356 enhanced the expression of ZO-1 and Occludin in HT-29 cells and Caco-2 monolayer cells models to reduce epithelial dysfunction caused by the enteroinvasive *Escherichia coli* (EIEC 029: NM) (43, 44). Similarly, our results indicated that *L. plantarum* G83 treatment at the medium and high doses increased the protein levels of ZO-1 and Occludin in the colon of DPMA mice. The effect of *L. plantarum* G83 on permeability varied by dose—the medium dose group had lower serum D-lactic acid whereas the high dose group had increased serum D-lactic acid—suggesting that other effects of *L. plantarum* G83 at high dose may offset the impact of increased tight junction proteins on permeability. Overall, these data demonstrate that *L. plantarum* G83 at a medium dose could be a candidate to increase colonic tight junction protein expression in the setting of colitis.

*L. plantarum* G83 treatment may also have alleviated colitis in DPMA mice through modulation of the gut microbiome, analogous to the effects of other probiotics that alter the intestinal microbiota through cross-feeding and inhibiting other species (45, 46). *L. plantarum* G83 showed promising probiotic properties in inhibiting pathogenic bacteria and improving beneficial bacteria in our previous studies (30). In this study, we found that the treatment of DPMA mice with *L. plantarum* G83 resulted in significant differences in microbial composition by beta diversity analysis at all three doses. This corresponded to reduced abundance of potential pathogenic bacteria, namely, *Enterorhabdus* spp., *Enterococcus* spp., *Klebsiella* spp., *Proteus* spp., *Providencia* spp., and *Veillonella* spp. after treatment with *L. plantarum* G83. Of these, infection with *Enterococcus* spp. is of particular relevance as multiple cases have been reported in captive pandas (47, 48). Moreover, panda-derived *Enterococcus* spp. exhibit acquired and intrinsic antibiotic resistance. Liu found that 15 isolates of panda-derived *Enterococcus* spp. carried many virulence genes and the detection rates of *ccf* and *efaAfs* reached 100%, while those of *gelE*, *ace*, and *efaAfm* were 66.7, 86.7, and 33.3%, respectively (49). Also, a recent study showed that *Proteus* spp. possessed many virulence factors potentially relevant to gastrointestinal pathogenicity, such as bacterial motility and adherence, the production of urease, hemolysins, and IgA proteases (50). In 2020, a *Proteus vulgaris* was isolated from the feces of an infected giant panda in the Fuzhou Zoo. This strain carried several virulent genes and showed resistance to 13 different drugs (51). FAPROTAX predicted function of the microbial community indicated that, compared with the BlankD group, the *L. plantarum* G83 treatment groups had less taxonomy related to the “human_pathogens_gastroenteritis” and “nitrate_reduction”. The decrease of “nitrate_reduction” may be due to a decrease in the abundance of *Enterobacteriaceae*, most of which could reduce the nitrate to nitrite (52). Our viable count and q-PCR result also supported that *L. plantarum* G83 could decrease absolute abundances of opportunistic pathogens within the *Enterobacteriaceae* family—including *E. coli*—in the panda-microbiota associated mice.

The *L. plantarum* G83 also showed an ability to promote other beneficial microbes, in particular an increased abundance of *Bifidobacterium* spp. in the medium and high dose groups. *Bifidobacterium* spp. have beneficial properties for the host health and can cross-feed other bacteria to produce short chain fatty acids (SCFA) (7, 53). SCFA have a wide range of beneficial effects such as promoting the epithelial barrier function, inhibiting inflammatory response, and supporting the metabolic requirements of colonic epithelium (54). *L. plantarum* G83 treatment increased the levels of acetic acid and butyric acid in the medium and high dose groups but not the low dose group, matching the pattern of *Bifidobacterium* expansion. Increased levels of two additional SCFA—propionic acid and valeric acid—was seen specifically in the medium dose group. This matched the higher predicted abundance of fermentation-related genes in this group (but not the other two dose groups) compared to the BlankD group. The induction of propionic acid specifically in the medium dose group could account for the increased serum levels of IL-10 specifically in the medium dose group, as prior human studies have found that propionic acid treatment increased serum IL-10 levels and peripheral regulatory T cells (55, 56). Thus, *L. plantarum* G83 supplementation may promote recovery in the DPMA mouse by promoting gut microbial changes that increase SCFA production.

Probiotics have been shown to affect the immune system through direct interaction with host immune cells or alteration of the microbiome (57–59). Pathogen-associated molecular patterns (PAMP) consisting of microbial components and metabolites are recognized by various pattern recognition receptors (PRRs), triggering the production of cytokines and chemokines that prime the immune system (60). Rocha-Ramírez demonstrated that direct exposure of inactivated *Lactobacillus* to macrophages successfully increased nuclear translocation of NF-κB-p65 and TLR2-dependent signaling, and induced proinflammatory cytokines such as IL-8, TNF-α, IL-12p70, and IL-6 (14). Conversely, *L. jensenii* TL2937 was shown to have the capability to inhibit TLRs by inducing expression of negative regulators of TLRs, including SIGIRR, A20, and IRAK-M, in CD172a^+^ cells (61). In our study, the *L. plantarum* G83 administration significantly increased TLR2, TLR4, and MyD88 gene expression. However, the downregulation of Iκk-β and NF-κB and the lower phosphorylation ratio of NF-κB in the TreatMG group were observed. To explore this contradiction, we further investigated the TLR-negative regulatory pathway. IL1RL1 can inhibit TLR-induced NF-κB activation by binding to MyD88 and MyD88 adapter-like (MAL) without affecting TRIF and IRAK. We found that the IL1RL1 gene was significantly increased in all three *L. plantarum* G83 treatment groups. This corresponded to reduced NF-κB-p65 phosphorylation in the medium and high dose groups. Considering the microbiota altered by *L. plantarum* G83 showed lower abundance pathogen associated taxa and higher beneficial bacteria, we speculate that *L. plantarum* G83 can not only restrain the inflammatory response by itself but also by altering intestinal microbiota.

Microbiota composition plays a crucial role in intestinal immune responses including the development of subpopulations of T lymphocytes (62). Specifically, the CD4^+^/CD8^+^ ratio is considered a marker for immune state that can be perturbed during inflammation and infection (63, 64). In our study, the CD4^+^/CD8^+^ ratio increased in the spleen, MLN, and PPs in the medium dose treatment group. This suggested that the immune system was activated by microbiota alteration in our study. Naïve T CD4^+^ cells can differentiate into T-helper (Th) subsets, namely, Th1, Th2, and Th17, each with distinct cytokine profiles that shape immune responses (65). It has been proposed that dysbiosis can contribute to inflammatory bowel diseases through alteration of the balance of Th1 or Th2 cells in the intestine and induction of Th17 responses. DSS colitis is characterized by a high ratio of Th2 cells, consistent with the previous report, and acute DSS colitis treatment caused significantly elevated levels of IL-6, TNF-α, and IL-17 (62). The low-dose *L. plantarum* G83 treatment group showed increased Th1/Th2 and Th1/Th17 ratios, suggesting that *L. plantarum* G83 could promote Th1 cells and/or inhibit the polarization of Th2 and Th17 cells (66, 67). The shifts in T helper subsets in the low dose treatment group corresponded to increased colonic sIgA secretion specifically in the low dose treatment group. Our findings are similar to those with VSL#3, a probiotic mixture which has been shown to induce Th1-type responses and suppress Th2 cytokine production (66). These results suggest that low dose *L. plantarum* G83 could regulate T helper balance, potentially through effects on the microbiota.

Altogether, our results revealed that *L. plantarum* G83 showed an ability to alleviate intestinal inflammation in DPMA mice which was associated with altered microbial composition, SCFA levels, intestinal immune cell activity, and T helper balance. These results support that *L. plantarum* G83 is a promising candidate to treat intestinal inflammation in panda.

## Data Availability Statement

The datasets presented in this study can be found in online repositories. The names of the repository/repositories and accession number(s) can be found in the article/**Supplementary Material**.

## Ethics Statement

The animal study was reviewed and approved by The Institutional Animal Care and Use Committee of Sichuan Agricultural University.

## Author Contributions

YZh and DL conceived and designed this experiment. YZh, YZe, JW, YP, and AK performed the experiments. YZh, DL, YZe, LN, CC, and JW performed the data analysis. YZh, DL, DZ and XN drafted the manuscript. JJ, KP, JF, BJ, DZ and XN helped to revise the manuscript. DZ and XN supervised all the experimental works. All authors contributed to the article and approved the submitted version.

## Funding

The research was supported by funding from the National Natural Science Foundation of China (31970503); the Project of Chengdu Giant Panda Breeding Research Foundation (CPF 2014-15) and the China Scholarship Council (201906910016).

## Conflict of Interest

The authors declare that the research was conducted in the absence of any commercial or financial relationships that could be construed as a potential conflict of interest.

## Publisher’s Note

All claims expressed in this article are solely those of the authors and do not necessarily represent those of their affiliated organizations, or those of the publisher, the editors and the reviewers. Any product that may be evaluated in this article, or claim that may be made by its manufacturer, is not guaranteed or endorsed by the publisher.
