# Supplementary material for: The Panda-Derived Lactobacillus plantarum G201683 Alleviates the Inflammatory Response in DSS-Induced Panda Microbiota-Associated Mice
Source: Front Immunol. 2021 Dec 8;12:747045. doi: 10.3389/fimmu.2021.747045 (PMC8692892; doi:10.3389/fimmu.2021.747045)
Supplement: Supplementary file 1 [file DataSheet_1.docx]

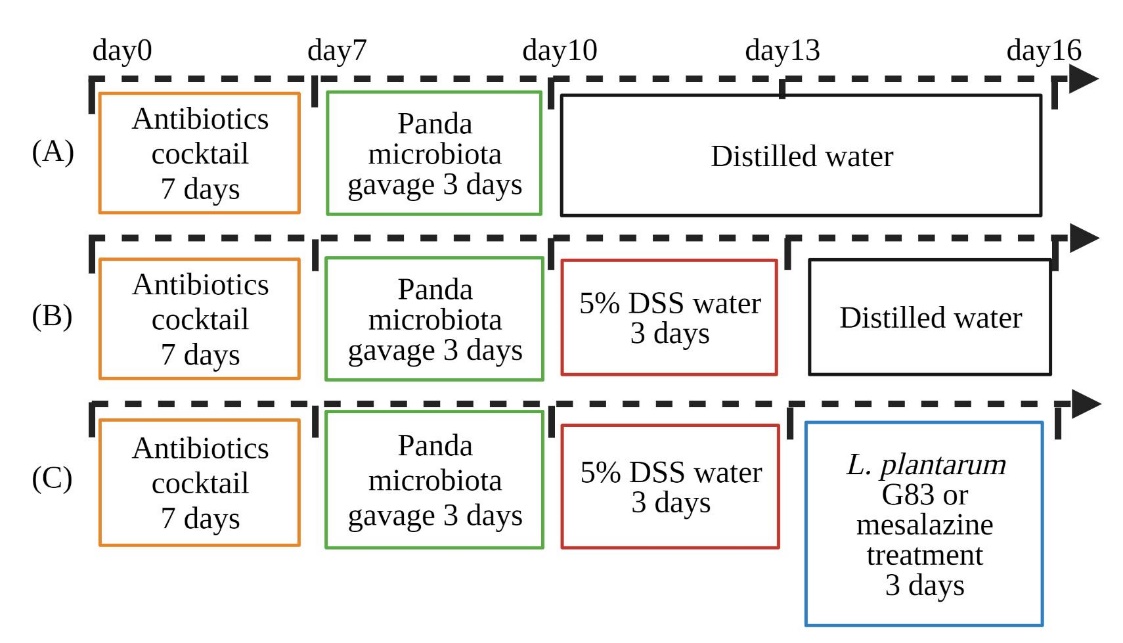


Supplementary figure 1 Animal trial and treatment design.

(A) Panda microbiota-associated (PMA) mice were generated by antibiotic administration. Antibiotic cocktail (600mg/L each of ampicillin, vancomycin, and neomycin) was administrated in the drinking water for 7 days, after which only distilled water was provided from day 7 to day 13. After antibiotic cocktail administration, mice were gavaged 0.2 ml/day panda feces microbiota inoculum 3 times (on day 7, 8, and 9). (B) DSS-induced panda microbiota-associated (DPMA) mice were generated by antibiotic administration followed by DSS drinking water. The same treatment as method (A) was used for the first ten days. Starting day 11, the mice were given 5% DSS drinking water for 3 days, after which only distilled water was provided from day 14 to day 16. (C) The DPMA mouse model was used in this study. Sixty mice were divided into 5 different groups equally, including the control group (no treatment received, BlankD), mesalazine treatment group (94mg/d, switched into the equivalent dose to the mouse, TreatMz group), and three 0.2ml/d *L. plantarum* G83 treatment groups (including, 1.0×10^6^ CFU/ml (TreatLG group), 1.0×10^7^ CFU/ml (TreatMG group), 1.0×10^8^ CFU/ml (TreatHG group)) from day 14 to day 16.


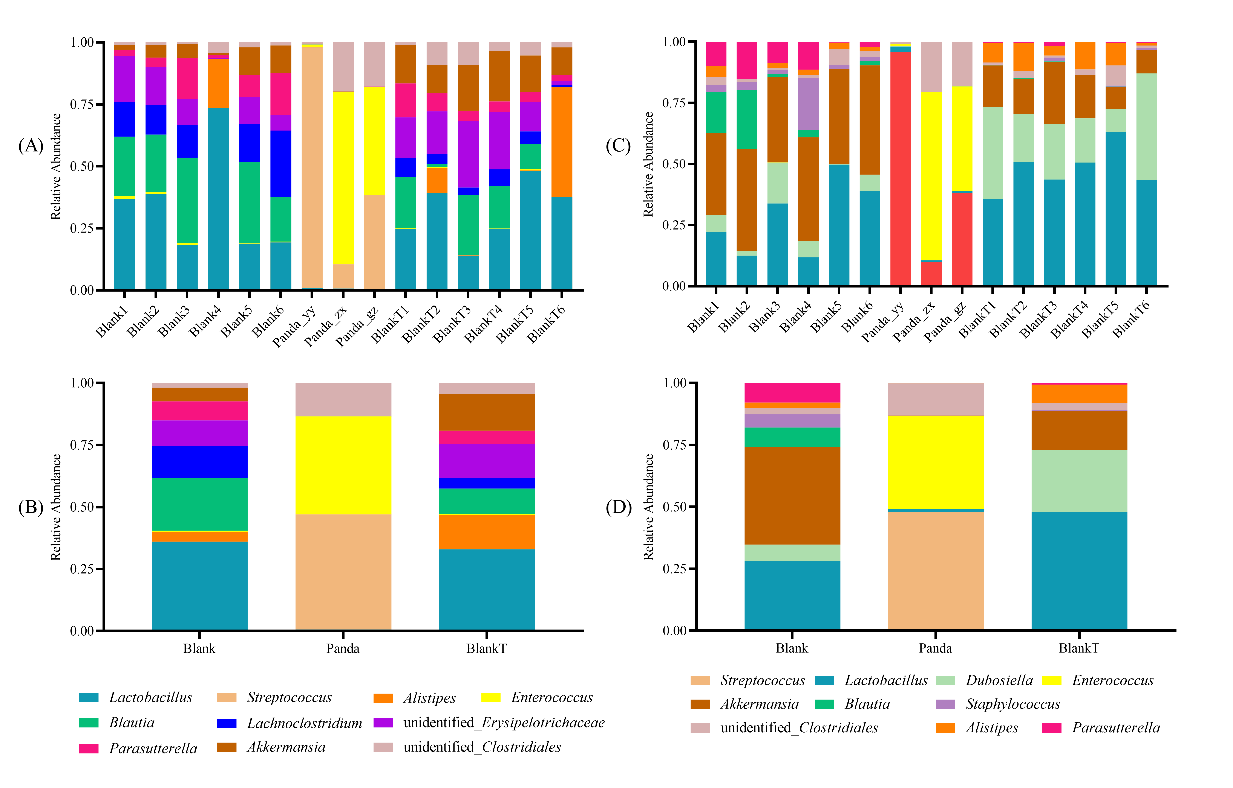


Supplementary figure 2 Taxonomic summary of the fecal microbiome at the genus level in the Blank, BlankT and Panda groups on day 13 and day 16.

Bar plots depicting the top 10 highest abundance bacterial genera by samples and groups on day 13 (A-B) and day 16 (C-D). Sample number: Blank, n=6; Panda, n=3; BlankT, n=6.


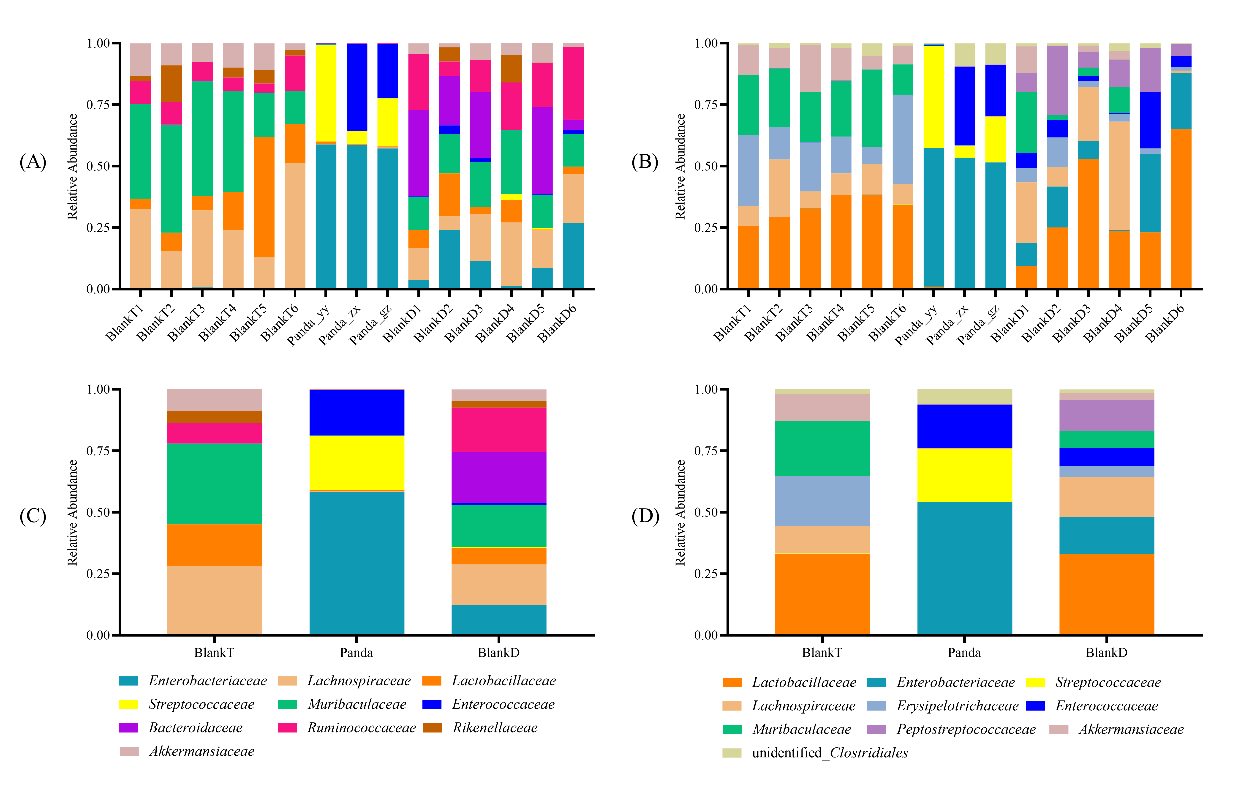


Supplementary figure 3 Taxonomic summary of the fecal microbiome at the family level in the BlankT, BlankD and Panda groups on day 13 and day 16.

Bar plots depict the top 10 highest abundance bacterial families by samples and groups on day 13 (A-B) and day 16 (C-D). Sample number: Blank, n=6; Panda, n=3; BlankT, n=6.


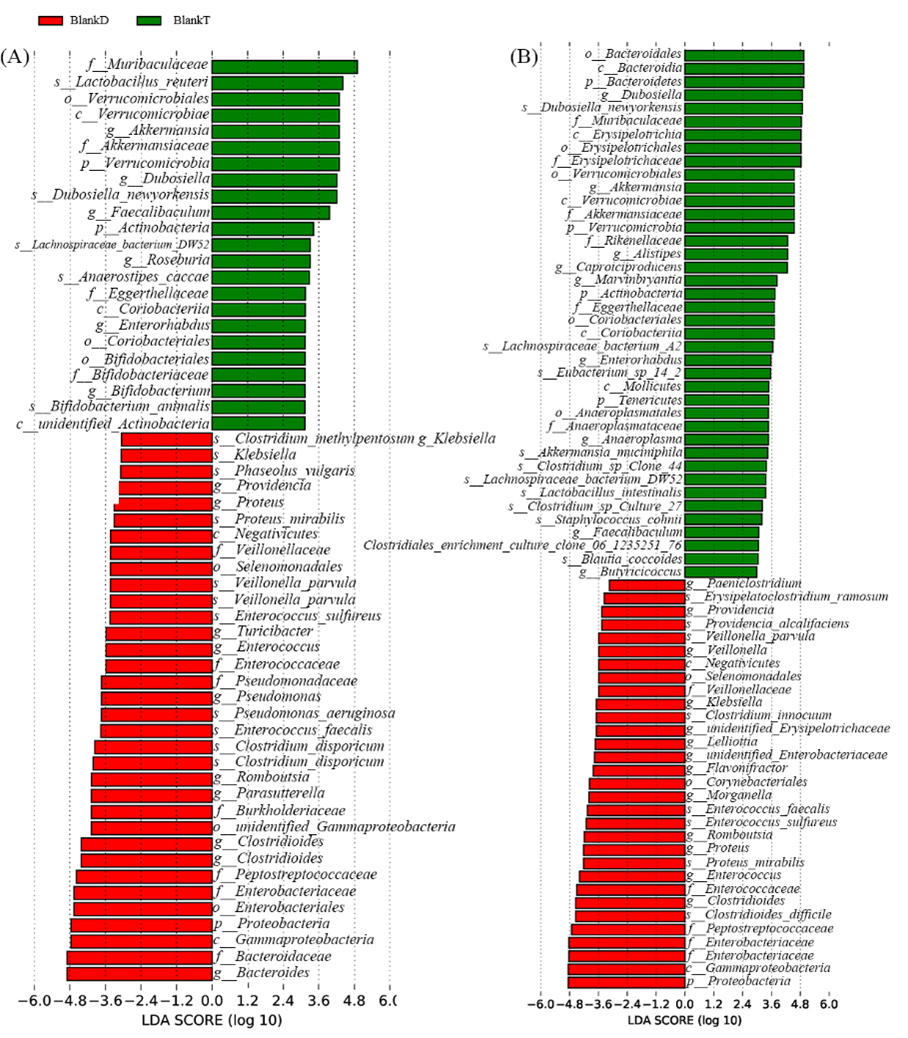


Supplementary figure 4 LEfSe analysis showing differentially abundant intestinal microbiota between the BlankT and BlankD groups on day 13 and day 16.

Linear discriminant analysis effect size (LEfSe) was performed to determine the marker genera that differed between the BlankT (n=6) and BlankD (n=6) groups on day 13 (A) and day 16 (B). The threshold for identifying differential genera was a logarithmic LDA score with magnitude greater than 2.0.


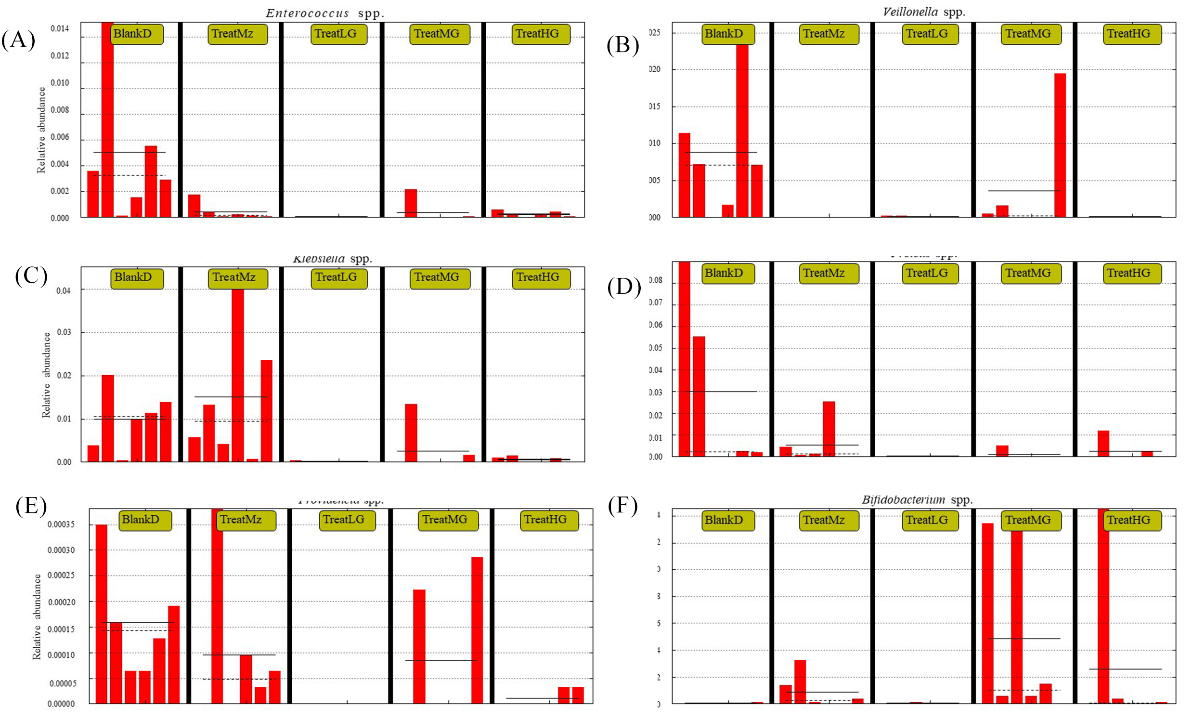


Supplementary figure 5 Relative abundances of microbial genera that were differentially abundant across treatment groups by LEfSe.

LEfSe was performed to identify differentially abundant DSS colitis marker genera. *L. plantarum* G83 treated groups showed lower abundance of *Enterococcus* spp. (A), *Veillonella* spp. (B), *Klebsiella* spp. (C), *Proteus* spp. (D), and *Providencia* spp. (E). These bacteria belong to the *Enterobacteriaceae* or *Enterococcaceae* families, which include opportunistic pathogens in the intestinal tract. The relative abundance of *Bifidobacterium* spp. (F) in the TreatMG and TreatHG groups was higher than that in the other groups.


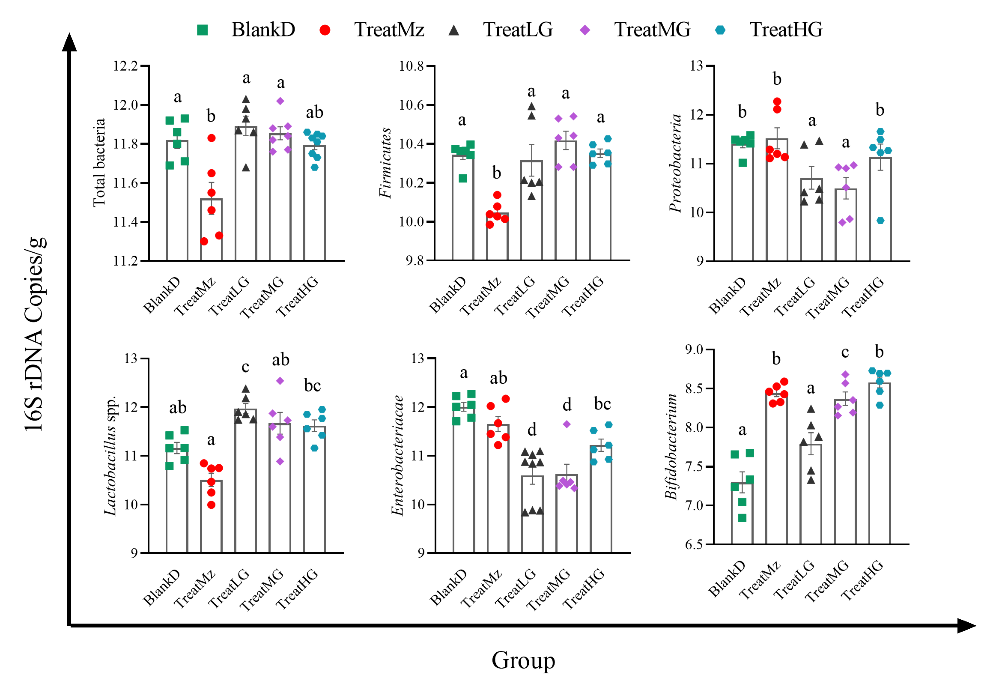


Supplementary figure 6 Absolute bacterial abundances in the luminal colon of DMPA mice.

DNA was extracted from colonic luminal content and underwent q-PCR with clade specific 16S rRNA primers to determine absolute abundance of various bacterial populations. The TreatMz group had decreased total bacteria and *Firmicutes* compared to the other groups. The *L. plantarum* G83 treatment groups had reduced abundance of *Proteobacteria* and *Enterobacteriaceae* compared to BlankD and TreatMZ. *Lactobacillus* spp. had higher abundance in the TreatLG group compared to the others and *Bifidobacterium* spp. had higher abundance in the TreatMz, TreatMG and TreatHG groups. All data are shown as mean ± SEM, n=6. Data were analyzed with one-way ANOVA (Tukey's test) and different letters mean p<0.05.


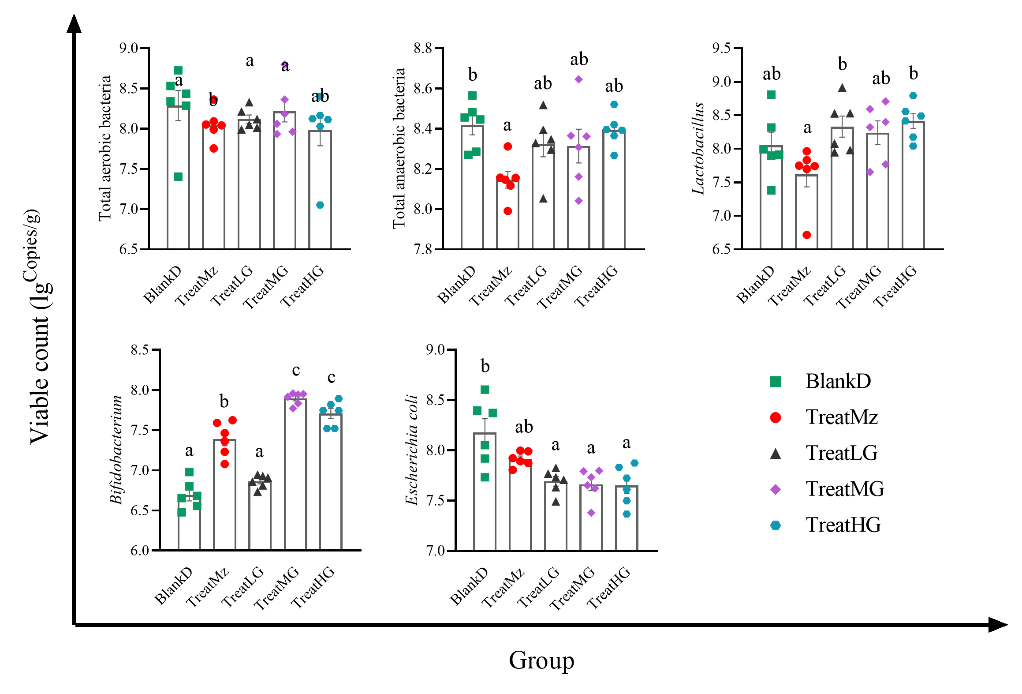


Supplementary figure 7 Viable bacterial counts in the luminal colon of DMPA mice.

The TreatMZ group had decreased total aerobic and anaerobic bacteria compared to the BlankD group. Higher abundance of *Bifidobacterium* spp. was observed in TreatMz, TreatMG and TreatHG groups compared to BlankD and TreatLG. *Escherichia coli*, which belongs to *Enterobacteriaceae*, was decreased in the luminal colon. All data are shown as mean ± SEM, n=6. Data were analyzed with one-way ANOVA (Tukey's test) and different letters mean p<0.05.


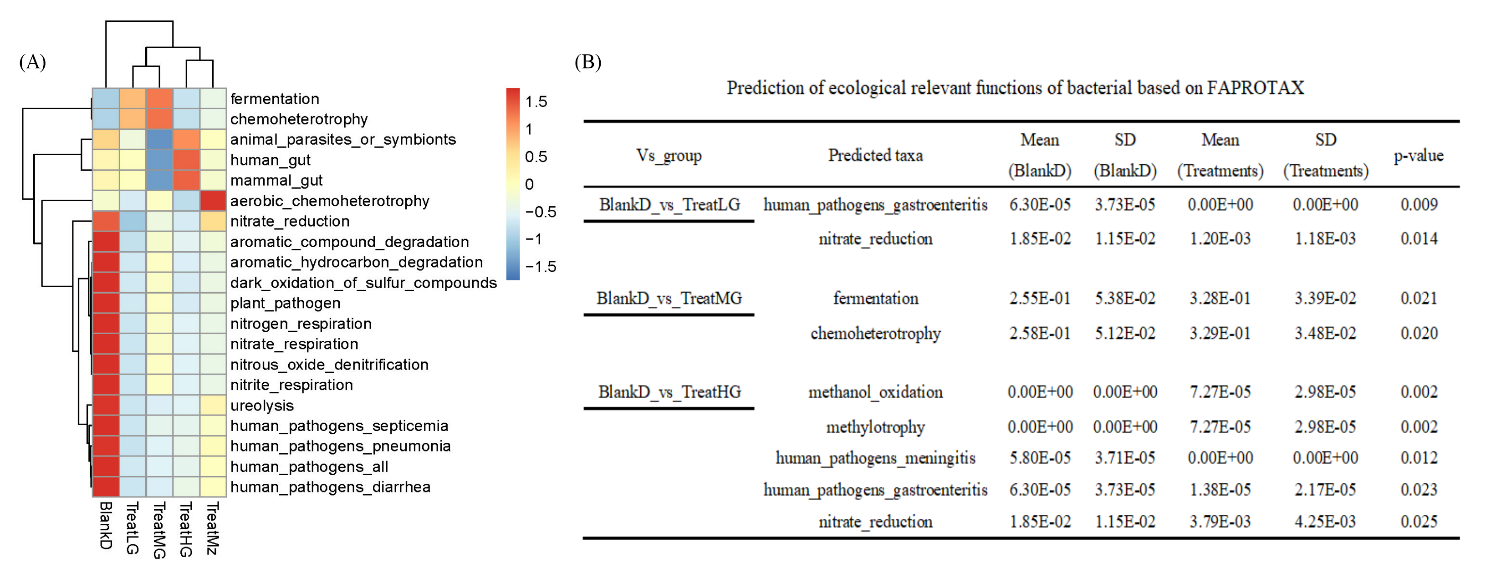


Supplementary figure 8 Prediction of ecologically relevant functions of intestinal bacteria.

FAPROTAX was used to predict ecologically relevant functions of bacterial from 16S rRNA sequencing data. Ecological relevant functions heatmap (A) showed that the BlankD group contained high abundance of human_pathogens relevant taxa. There was lower abundance of human_pathogens and nitrate_reduction relevant taxa in the TreatLG and the TreatHG groups compared to the BlankD group. Higher abundance of fermentation relevant taxa was observed in the TreatMG group compared with the BlankD group. No different taxa were observed between BlankD and TreatMz group.
